# Control of Oocyte Reawakening by Kit

**DOI:** 10.1371/journal.pgen.1006215

**Published:** 2016-08-08

**Authors:** Hatice Duygu Saatcioglu, Ileana Cuevas, Diego H. Castrillon

**Affiliations:** Department of Pathology and Cecil H. and Ida Green Center for Reproductive Biology Sciences, UT Southwestern Medical Center, Dallas, Texas, United States of America; University of Wisconsin Madison School of Veterinary Medicine, UNITED STATES

## Abstract

In mammals, females are born with finite numbers of oocytes stockpiled as primordial follicles. Oocytes are “reawakened” via an ovarian-intrinsic process that initiates their growth. The forkhead transcription factor Foxo3 controls reawakening downstream of PI3K-AKT signaling. However, the identity of the presumptive upstream cell surface receptor controlling the PI3K-AKT-Foxo3 axis has been questioned. Here we show that the receptor tyrosine kinase Kit controls reawakening. Oocyte-specific expression of a novel constitutively-active *Kit*^*D818V*^ allele resulted in female sterility and ovarian failure due to global oocyte reawakening. To confirm this result, we engineered a novel loss-of-function allele, *Kit*^*L*^. *Kit* inactivation within oocytes also led to premature ovarian failure, albeit via a contrasting phenotype. Despite normal initial complements of primordial follicles, oocytes remained dormant with arrested oocyte maturation. Foxo3 protein localization in the nucleus versus cytoplasm explained both mutant phenotypes. These genetic studies provide formal genetic proof that Kit controls oocyte reawakening, focusing future investigations into the causes of primary ovarian insufficiency and ovarian aging.

## Author Summary

In mammals, oocyte reawakening controls female fertility, the onset of the menopause, and thus, overall aging. We demonstrate here through complementary genetic experiments that Kit is the upstream receptor regulating oocyte reawakening. Although other cell surface receptors have been proposed as candidates, the data have remained contradictory, and definitive genetic evidence in support of any one receptor has been lacking. We engineered two novel Kit alleles in mice, one an activating (gain-of-function) mutation, the other an inactivating (loss-of-function) mutation. These alleles permitted us to conduct elegant genetic experiments whereby Kit was activated or inactivated in the oocytes of newborn mice. The results were complementary and striking. Oocyte-specific Kit activation resulted in female sterility due to reawakening of all oocytes, leading to premature ovarian failure. In contrast, Kit inactivation also led to female sterility and ovarian failure, but through a contrasting and opposite phenotype: a complete failure of primordial follicle reawakening. Additional studies demonstrated that Foxo3, a known regulator of reawakening, was the mediator of both phenotypes, linking our findings to prior discoveries. These complementary genetic experiments thus definitively incriminate Kit as the upstream receptor regulating reawakening.

## Introduction

**Fig 1:**
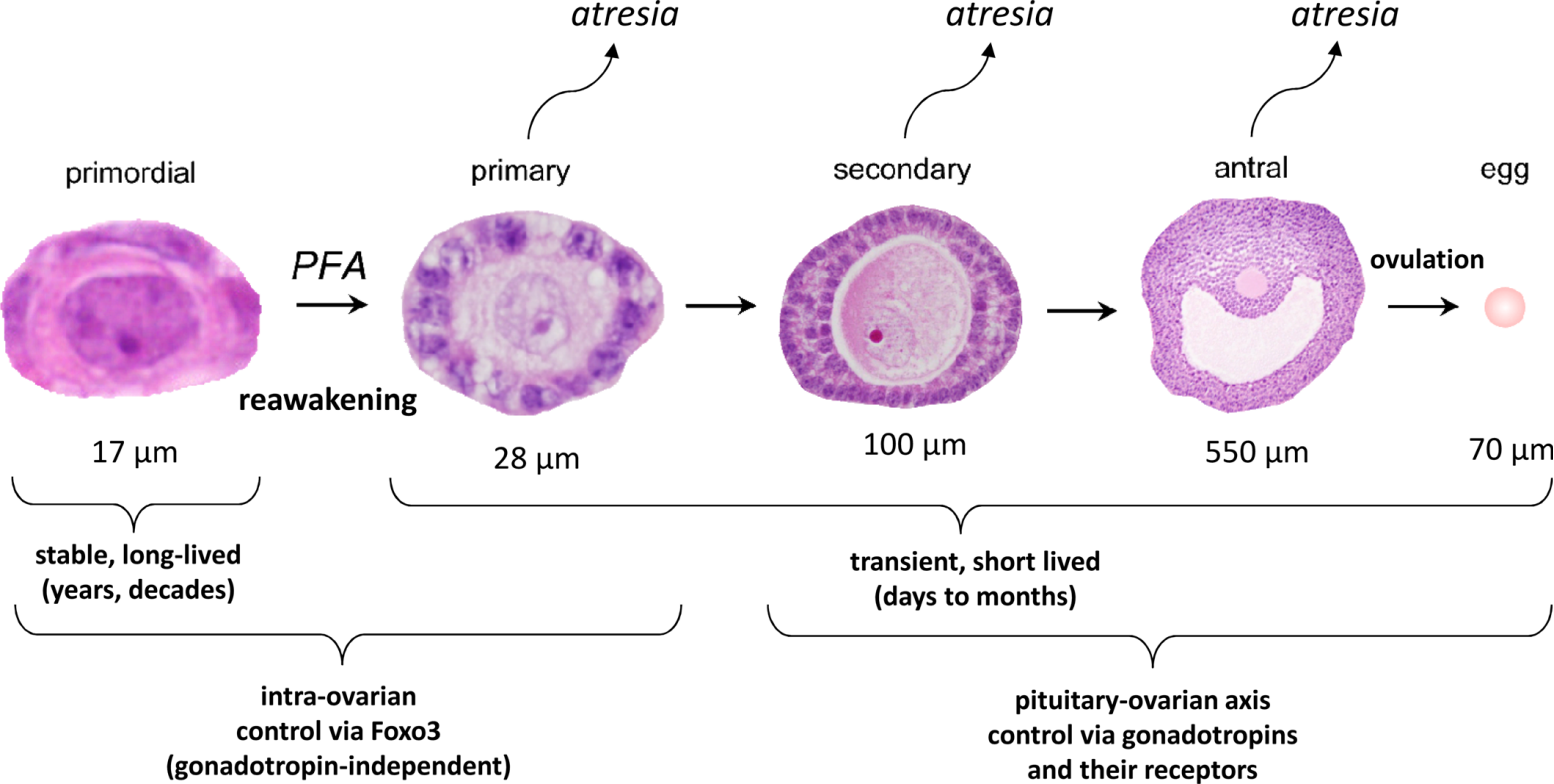
Stages of follicular maturation. Mouse follicles are depicted (not to scale: approximate follicle sizes including granulosa cells are shown below each follicle). Follicle growth is normally asynchronous, such that the adult ovary contains follicles at all stages of development. However, the vast majority of follicles at any time are primordial, with only a very small percentage of follicles actively growing. Follicle reawakening, sometimes called initiation or activation, is the regular, metered process by which individual primordial follicles are continually selected to begin the elaborate program of follicle maturation that culminates in ovulation. The earliest morphologic hallmark of reawakening is increased oocyte size followed by a change in granulosa cells from a flattened to a cuboidal shape, and then by granulosa cell proliferation. Whereas primordial follicles are long-lived, growing follicles are transient and short-lived, because growing follicles that do not progress to the next stage undergo atresia. Primary follicles are defined as follicles that have initiated oocyte growth and undergone a transition from flattened to cuboidal granulosa cells but have only one layer of granulosa cells. Secondary follicles have two layers of granulosa cells, and antral follicles are large follicles with a chamber (antrum) in preparation for ovulation.

Primordial follicles are the reserve precursor pool for maturing follicles throughout reproductive life [1]. Primordial follicles are reawakened via an ovarian-intrinsic (gonadotropin independent) process whereby they are selected from the quiescent reserve into the growing follicle pool [2, 3]. The morphologic hallmark of reawakening is oocyte growth, and this is followed by a transition of the surrounding granulosa cells from a flattened to a cuboidal shape [4]. Reawakening is irreversible, in that follicles that have initiated growth undergo atresia if not selected for subsequent stages of maturation. Primordial follicle numbers decrease with advancing age due to oocyte reawakening or apoptosis; following follicle depletion, ovulation ceases and reproductive senescence ensues. Reawakening must therefore be metered throughout reproductive life to ensure that some growing follicles are available during each estrus cycle, but at the same time, limit the number of growing follicles to forestall depletion of primordial follicles (see Fig 1 for summary schematic of follicle maturation). Characterization of the molecular mechanisms underlying reawakening remains an important challenge in reproductive biology [5–8].

The forkhead transcription factor Foxo3 functions as a switch that controls (suppresses) reawakening. In nullizygous or oocyte-conditional *Foxo3* knockout mice, primordial follicles are assembled normally [9], but undergo global reawakening at birth. This leads to a characteristic sequential syndrome of ovarian hyperplasia, follicle depletion, and hypergonadotropic ovarian failure [10]. In the adult ovary, Foxo3 protein localizes to the primordial oocyte nucleus, where it restrains reawakening in a PI3K (phosphoinositide-3-kinase)-AKT dependent manner. PI3K catalyzes the formation of a lipid second messenger, phosphatidylinositol 3,4,5-trisphosphate (PIP3), from phosphatidylinositol 4,5-bisphosphate (PIP2). PIP3 in turn leads to the phosphorylation and activation of AKT. Pten, a lipid phosphatase that converts PIP3 back to PIP2, potently suppresses AKT activation [11]. Oocyte-specific deletion of *Pten* hyperactivates AKT, resulting in Foxo3 nuclear export and global reawakening [12, 13]. Foxo3 normally undergoes nuclear export during the primordial to primary follicle transition (followed by its degradation within the cytoplasm via unknown mechanisms), suggesting that *Pten* inactivation mimics a naturally-occurring PI3K-dependent signal that regulates Foxo3 localization and hence reawakening [13]. Other studies have confirmed Foxo3’s role as the molecular switch controlling reawakening in a PI3K-AKT dependent manner, and the utility of Foxo3 nuclear vs. cytoplasmic localization as a marker of oocyte maturation [14–18]. Oocyte-specific inactivation of the mTOR inhibitors *Tsc1* and *Tsc2* also results in oocyte reawakening, establishing an important but incompletely understood role of mTOR signaling in this process [19].

As vital effectors of PI3K-AKT signaling, the Foxos serve fundamental biological roles in aging, cancer, and stem cell maintenance [20–22]. The three canonical Foxos—Foxo1, Foxo3, and Foxo4—are coexpressed and exhibit genetic and functional redundancy in most cell types [20, 21, 23]. In contrast, in the mouse germline, Foxo1 and Foxo3 have diverged to serve complementary roles in the maintenance of the male and female germline, respectively. Whereas Foxo3 is the principal Foxo protein in the oocyte, Foxo1 is the principal Foxo within undifferentiated spermatogonia, and is the only Foxo required for the maintenance of spermatogonial stem cells [20, 24]. In both the female and male germline, genetic experiments have shown that Foxo activity is regulated by its subcellular (nuclear vs. cytoplasmic) localization via its AKT-dependent phosphorylation. When phosphorylated, Foxo proteins are functionally inhibited by their retention in the cytoplasm via interactions with 14-3-3 proteins [25].

In many physiological processes, the upstream activators of class I PI3Ks are transmembrane receptor tyrosine kinases (RTKs) such as Igf1r, insulin receptor, Ret, Pdgfr, or Kit [11]. Ligand binding activates PI3K by phosphotyrosine-mediated binding through an SH2 domain on the p85 subunit of PI3K. G protein–coupled receptors (GPCRs) can also activate PI3K through the p110γ catalytic subunit isoform. However, p110γ^−/−^ mice are viable and fertile (but display various GPCR-mediated immunological defects), suggesting that GPCRs may not play essential roles in the regulation of the PI3K pathway during oocyte reawakening [26]. The identification of a presumptive oocyte surface RTK that acts through PI3K-AKT-Foxo3 to regulate reawakening has remained an outstanding question in reproductive biology [27]. Several candidates including Kit have been proposed, but definitive evidence about which is the *bona fide* receptor has been lacking [3, 8, 28–30]. The unique biological features of primordial follicle reawakening, some of which are not readily modeled *in vitro*, prompted us to apply genetic approaches to identify and validate this factor [3].

## Results

### Generation and validation of the conditional dominant gain-of-function allele *Kit*^*D818V(L)*^

Kit is an RTK that acts via PI3K-AKT, and is expressed within primordial oocytes [31, 32]. To study the role of Kit in the reawakening of primordial follicles, we generated a novel murine conditional allele, *Kit*^*D818V(L)*^ through homologous recombination in murine embryonic stem cells. The Kit D818V (Asp→Val) amino acid substitution leads to constitutive Kit activity in the absence of ligand (KL), and exerts potent dominant gain-of-function effects [31, 33]. Furthermore, it corresponds to the most common known *Kit* gain-of-function mutation in human germ cell tumors (*Kit*^*D816V*^), demonstrating that this mutant protein is active in germ cells [33–35]. The conditional (floxed) *Kit*^*D818V(L)*^ allele was designed to provide Kit function through a 3’ cDNA cassette encoding exons 17–21. Cre-mediated recombination excises this cDNA cassette, permitting normal splicing of an exon 17 harboring the mutation, and thus expression of mutant D818V protein (S1A Fig). With respect to nomenclature for the four new *Kit* alleles described in this manuscript, 1) genotypes signify somatic genotypes (per tail DNA genotyping) and 2) the floxed (i.e., latent) alleles end in “(*L*)”. Whereas hemizygous *Kit* (a.k.a. *Dominant white spotting*) loss-of-function mutations produce abnormal coat pigmentation [36] (see also below), mice harboring the *Kit*^*D818V(L)*^ allele were externally normal with coat pigmentation similar to sibling controls, confirming that the floxed allele indeed provided Kit function (S1B Fig). The *Kit*^*D818V(L)*^ allele could be homozygosed, and such animals were also externally indistinguishable from littermate controls (S1C Fig).

### *Kit* activation in primordial oocytes results in a global primordial follicle reawakening phenotype

**Fig 2:**
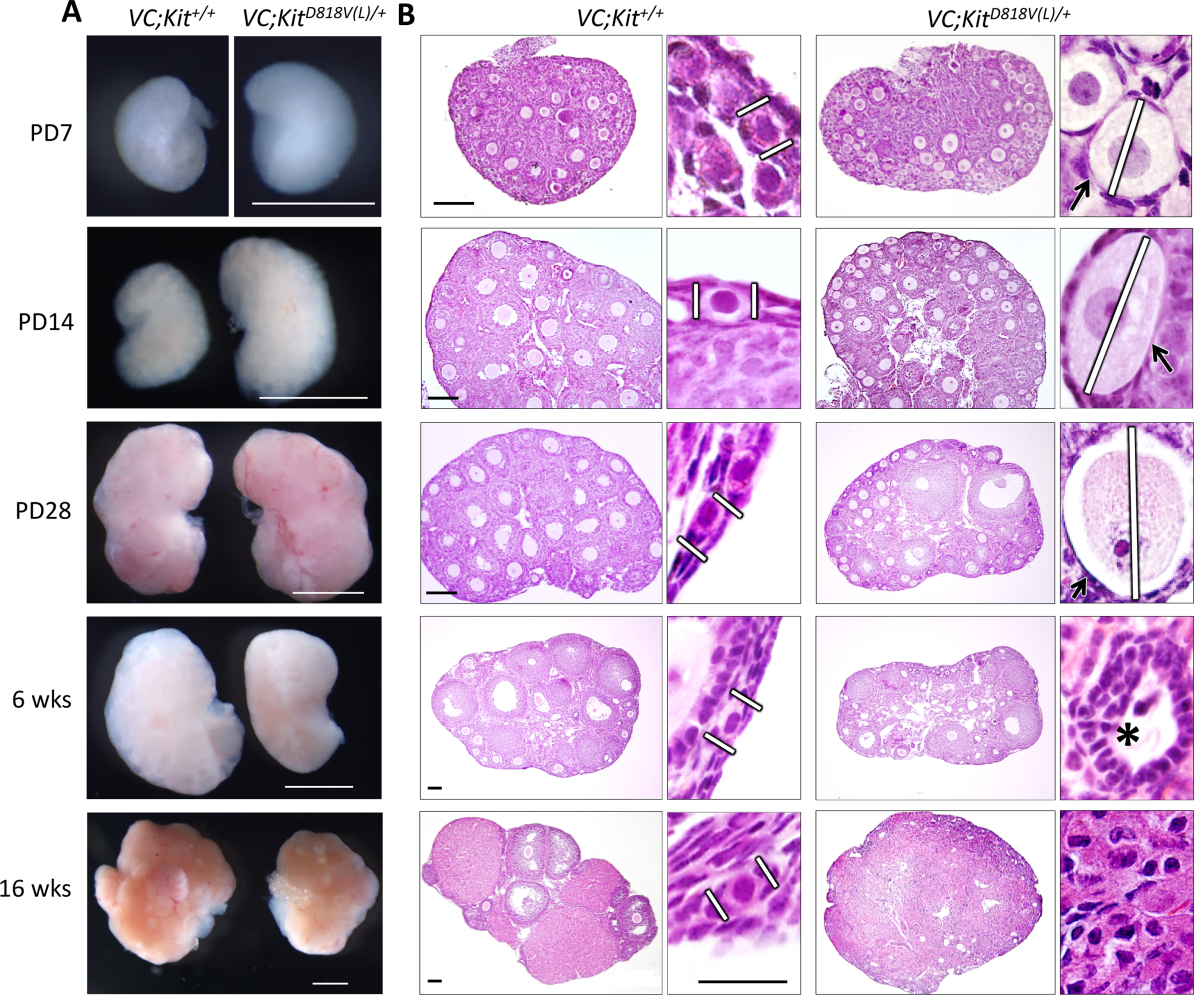
Global oocyte reawakening phenotype following conditional Kit activation within oocytes. (A) *VC*; *Kit*^*D818V(L)*^*/+* ovaries were larger than sibling controls up to PD28; scale bars = 1 mm. (B) Histological analyses (H&E-stained sections) revealed significant oocyte enlargement (white lines) and flattened granulosa cells (arrows) in many of the activated follicles (PD7-28). Numerous preantral and early antral follicles are present at PD28. By 6 weeks there was some oocyte death, follicle atresia and empty follicles (asterisk); by 16 weeks there was a complete absence of follicles with the ovary consisting of a luteinized stroma. These features indicate a classic global reawakening phenotype. Scale bars = 100 μm in large panels (same for control and experimental sections); 25 μm for small panels (all at the same magnification). Pictures are representative of at least three animals analyzed per genotype/time-point.

These mice were then bred to the germ cell-specific Cre driver, *Vasa-Cre* (a.k.a. *Ddx4-cre*^*1Dcas/J*^) (abbreviated *VC*) to generate *VC*; *Kit*^*D818V(L)*^*/+* females. *VC* becomes active during late embryogenesis, and drives Cre-mediated recombination in >99% of oocytes by birth [37]. Ovaries were harvested at postnatal day (PD) 7 and ovarian cDNA was analyzed by RT-PCR, followed by Sanger sequencing. As expected, ovaries from experimental females expressed mutant cDNA at levels close to wild-type as evidenced by electrophoretogram peak intensities (S1D Fig). By PD7, *VC*; *Kit*^*D818V(L)*^*/+* ovaries were consistently larger than ovaries from sibling controls. By PD14, these size differences were even more marked, but from PD28 onward (up to 16 weeks of age), ovaries were of equal size or somewhat smaller (Fig 2A). To understand the cellular basis of this increase in ovarian size (and subsequent decrease), tissue sections were analyzed. At PD7, there was an obvious increase in oocyte diameters in follicles that otherwise resembled primordial follicles (i.e., follicles without granulosa cell growth or change to cuboidal shape) (Fig 2B). Interestingly, whereas Kit protein is predominantly membranous in controls, Kit protein underwent a general redistribution to the cytoplasm in *VC*; *Kit*^*D818V(L)*^*/+* oocytes (S2A–S2C Fig) with overall Kit protein levels comparable to *Kit*^*D818V(L)*^*/+* controls (S2D Fig). This is consistent with prior data demonstrating that Kit protein normally undergoes ligand-dependent internalization, and that constitutively active mutant variants are internalized more efficiently than the wild-type protein [38, 39].

Of note, these morphological alterations were global, occurring in all primordial follicles. *VC*; *Kit*^*D818V(L)*^*/+* oocytes grew in size up to PD28, resulting in aberrant follicles with dramatically enlarged oocytes (Figs 2B and S4A). Some morphologically normal follicles and corpora lutea stage were also present, indicating that some of the reawakened follicles progressed normally to more advanced states of follicle maturation. Concordantly, most markers of primordial or primary follicles including periodic acid-Schiff (PAS) stain (labels the zona pellucida), ZP1, Inhibin, Gdf9, Sohlh1, Nobox, and Sall4 retained their typical patterns of expression in oocytes or granulosa cells, consistent with normal differentiation despite global reawakening (S3 and S4A Figs). However, in some reawakened follicles, granulosa cells remained flattened and were negative for α-Müllerian Hormone (AMH), which is normally induced at the primary follicle stage (S4A Fig). A similar spectrum of abnormalities has been documented in other global reawakening mutants such as *Foxo3* and *Pten* [10, 12, 13].

By 16 weeks, however, oocyte atresia occurred, resulting in morphologically abnormal, “empty” follicles depleted of oocytes (note also the absence of more advanced follicles and corpora lutea) (Fig 2B). No teratomas or other ovarian tumors were identified. Thus, constitutive Kit activation in the female germline resulted in a classic, global primordial follicle reawakening phenotype identical to that described for *Foxo3* and *Pten* [10, 12, 13]. Global reawakening occurred in all *VC*; *Kit*^*D818V(L)*^*/+* primordial oocytes, which ultimately underwent atresia resulting in loss of all oocytes with premature ovarian failure. Serum follicle stimulating hormone (FSH) and luteinizing hormone (LH) levels at 5 months of age were elevated in adult mutant females, consistent with hypergonadotropic hypogonadic premature ovarian failure (P<0.003 and P<0.02 respectively) (S4B Fig). These results strongly implicate Kit as the upstream RTK regulating primordial oocyte reawakening.

**Fig 3:**
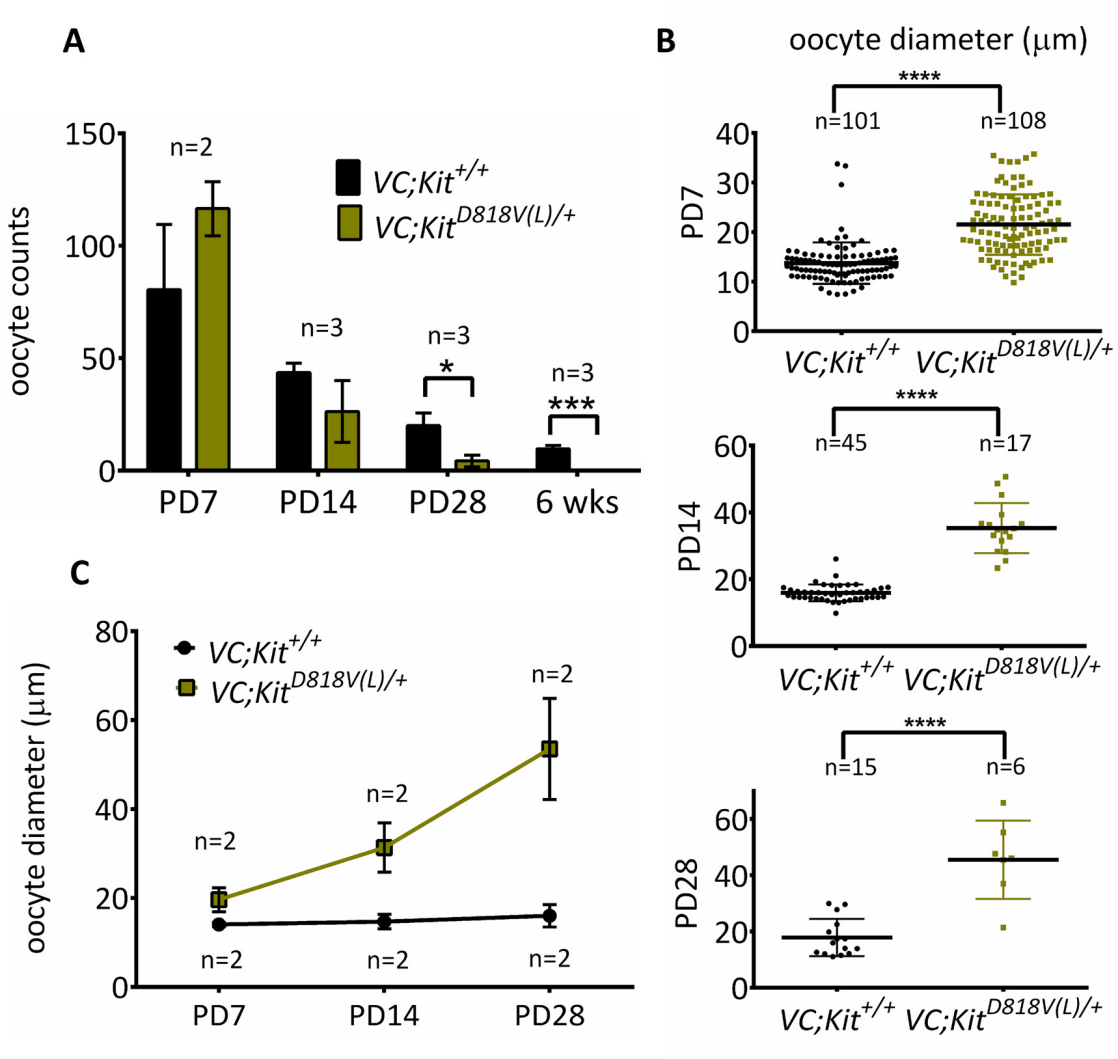
Quantitative analyses of oocyte phenotypes following conditional Kit activation within oocytes. (A) Primordial/primary oocyte numbers of *VC*; *Kit*^*D818V(L)*^*/+* and control ovaries (n≥2 animals per genotype per timepoint; *p<0.05; ***p<0.001, unpaired student t test, error bars = S.E.M. (B) Oocyte diameters (micrometers) of early oocytes in *VC; Kit*^*D818V(L)*^*/+* and *VC; +/+* mice; ****p<0.0001, n≥6 unpaired student t test. Error bars are S.E.M. (C) Average primordial/primary oocyte diameters in *VC; Kit*^*D818V(L)*^*/+* and *VC; +/+* ovaries; at least 6 oocytes were measured from two different animals per genotype/time-point.

At birth to PD7, oocyte numbers were unaltered in *VC*; *Kit*^*D818V(L)*^*/+* ovaries, indicating a normal initial endowment of oocytes [3]. Oocytes were markedly depleted by PD28, and totally absent by 6 weeks of age (P<0.02 and P<0.0004, respectively) (Fig 3A). Measurements of oocyte diameters confirmed that average oocyte sizes were significantly increased by PD7, and the difference was even more marked at PD14 (P<0.0001 for both PD7 and PD14). Oocytes continued to grow through PD28, although by this timepoint, relatively few oocytes remained (Figs 3B, 3C and S4A). Sections from experimental and control ovaries embedded in plastic confirmed the absence of oocytes by 6 weeks of age as well as the presence of atretic oocytes and zona pellucida remnants (the “ghosts” of oocytes that underwent reawakening and subsequent atresia) in *VC*; *Kit*^*D818V(L)*^*/+* females (S4C Fig).

### *Kit* activation results in global primordial oocyte reawakening via AKT-Foxo3

**Fig 4:**
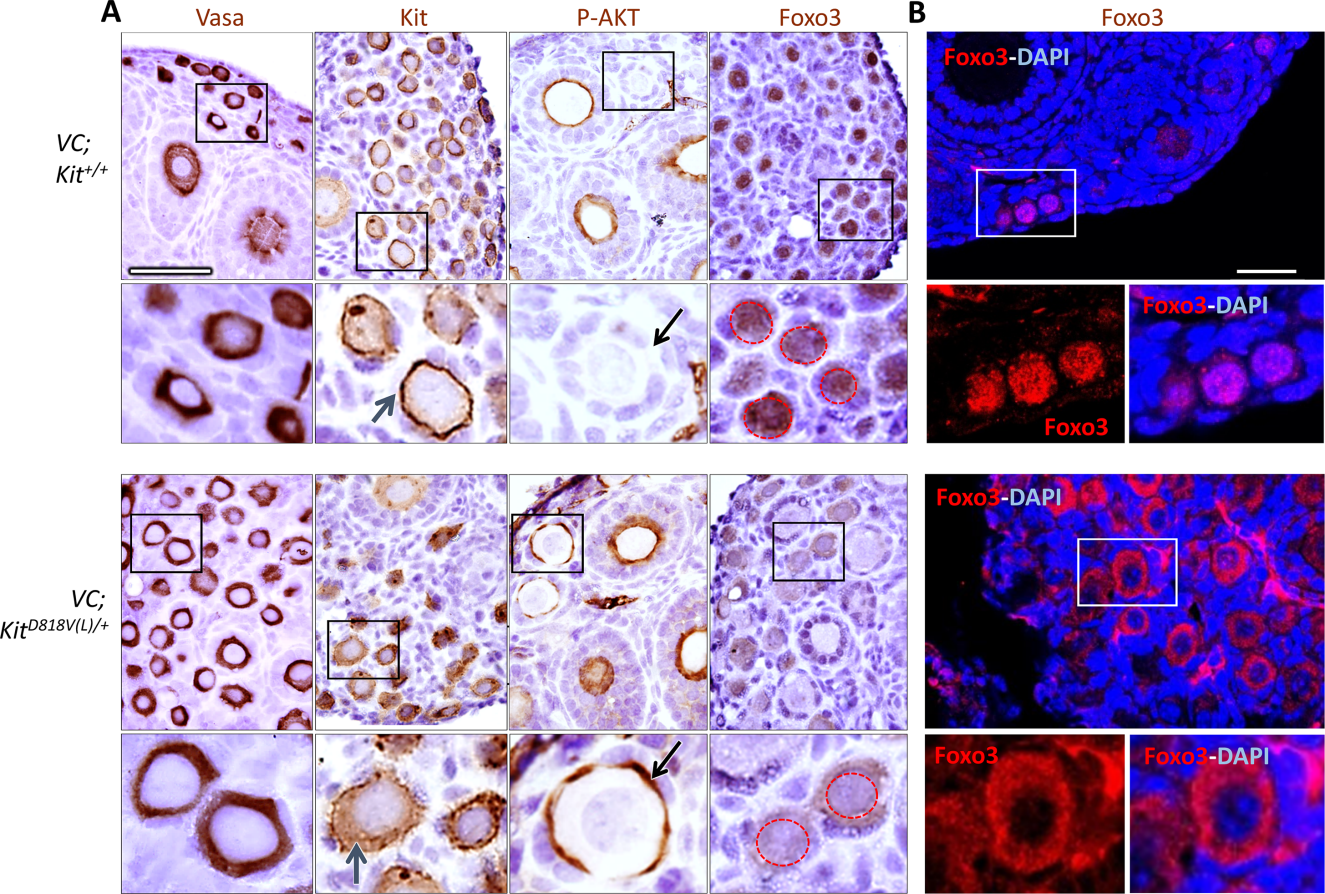
Kit activation promotes oocyte reawakening through AKT-Foxo3 axis. (A) Immunohistochemistry for markers as shown; slides counterstained with hematoxylin. Lower panels are higher magnifications of areas shown in black rectangles. Blue arrows, Kit localization in experimental and control oocytes. Note membrane-bound staining in the control vs. greater cytoplasmic staining in *VC; Kit*^*D818V(L)*^*/+* oocytes. Black arrows, oocyte membrane of primordial/primary follicles in control vs. mutant; only mutant oocytes were positive for P-AKT. Red dashed circles in Foxo3 panels demarcate nuclei to highlight nuclear to cytoplasmic export in the mutant. Scale bar = 25 μm, all panels at same magnification. (B) Foxo3 localization by confocal microscopy/immunofluorescence of control and experimental ovaries at PD14. Scale bar = 33 μm for both large panels. Lower panels are higher magnifications of areas in white rectangles.

Next, various markers were analyzed by immunohistochemistry at PD*7* to further characterize potential abnormalities in downstream effectors such as Foxo3. All oocytes were strongly Vasa-positive, indicating preservation of germline identity following Kit activation. Kit protein was present at high levels within oocytes, although some redistribution to the cytoplasm was evident in the mutant as was the case by immunofluorescence (Fig 4A). Activated early oocytes exhibited increased phosphorylated AKT (P-AKT) on their cell membranes as compared to control primordial and primary follicles, which did not contain detectable P-AKT (Fig 4A). This is consistent with the known role of PI3K-AKT as the key signaling pathway mediating oocyte reawakening [13], and argues strongly that Kit controls reawakening via this pathway. Importantly, AKT hyperphosphorylation drove translocation of Foxo3 protein from the nucleus to the cytoplasm in all *VC*; *Kit*^*D818V(L)*^*/+* oocytes (Fig 4A). Confocal microscopy, which allows for higher resolution than immunohistochemistry, confirmed these results; i.e. Foxo3 was predominantly nuclear in controls, but cytoplasmic in the mutant oocytes (Fig 4B).

### Generation and validation of conditional floxed allele *Kit*^*L*^

The above results provided strong genetic evidence implicating Kit as the upstream RTK controlling oocyte reawakening via the PI3K-AKT-Foxo3 axis. However, phenotypes associated with gain-of-function mutations should generally be interpreted with caution, as even a single amino acid substitution could have multiple, distinct, and potentially unexpected effects on protein function and thus, phenotypes. To expand upon our genetic analyses of *Kit* in oocyte reawakening with a complementary genetic approach, we designed a new allele where exon 17, which encodes the kinase domain [31], was floxed. *Kit* is an essential locus due to its requirement for hematopoiesis, necessitating conditional genetic analysis. The floxed allele *Kit*^*L*^ could be homozygosed as expected, and *Kit*^*L*^*/Kit*^*L*^ animals were born at expected Mendelian ratios (S5A and S5B Fig). For oocyte-specific *Kit* inactivation, *VC*; *Kit*^*L/+*^ fathers were bred to *Kit*^*L*^*/Kit*^*L*^ females. *VC* activity in the father’s germline converts any paternally transmitted *Kit*^*L*^ allele to *Kit*^*-*^ and thus, *VC* fathers can transmit a wild-type (*Kit*^*+*^) or null *Kit*^*-*^allele but not a *Kit*^*L*^ allele [37]. Experimental females of the *VC*; *Kit*^*L/-*^ genotype (per tail DNA) thus harbor homozygous *Kit*^*-*^ loss-of-function mutations in their germline. RT-PCR of ovaries (S5C Fig) and Sanger sequencing of PCR-amplified cDNAs (S5D Fig) confirmed *VC*-dependent deletion of exon 17. Whereas control mice harboring the floxed allele exhibited no pigmentation or other external abnormalities, *VC*; *Kit*^*L/-*^ mice showed striking midline hypopigmentation, consistent with hemizygous somatic loss of Kit activity (S5E Fig).

### Conditional inactivation of *Kit* within primordial oocytes results in a complete failure of oocyte reawakening with persistence of quiescent primordial follicles

**Fig 5:**
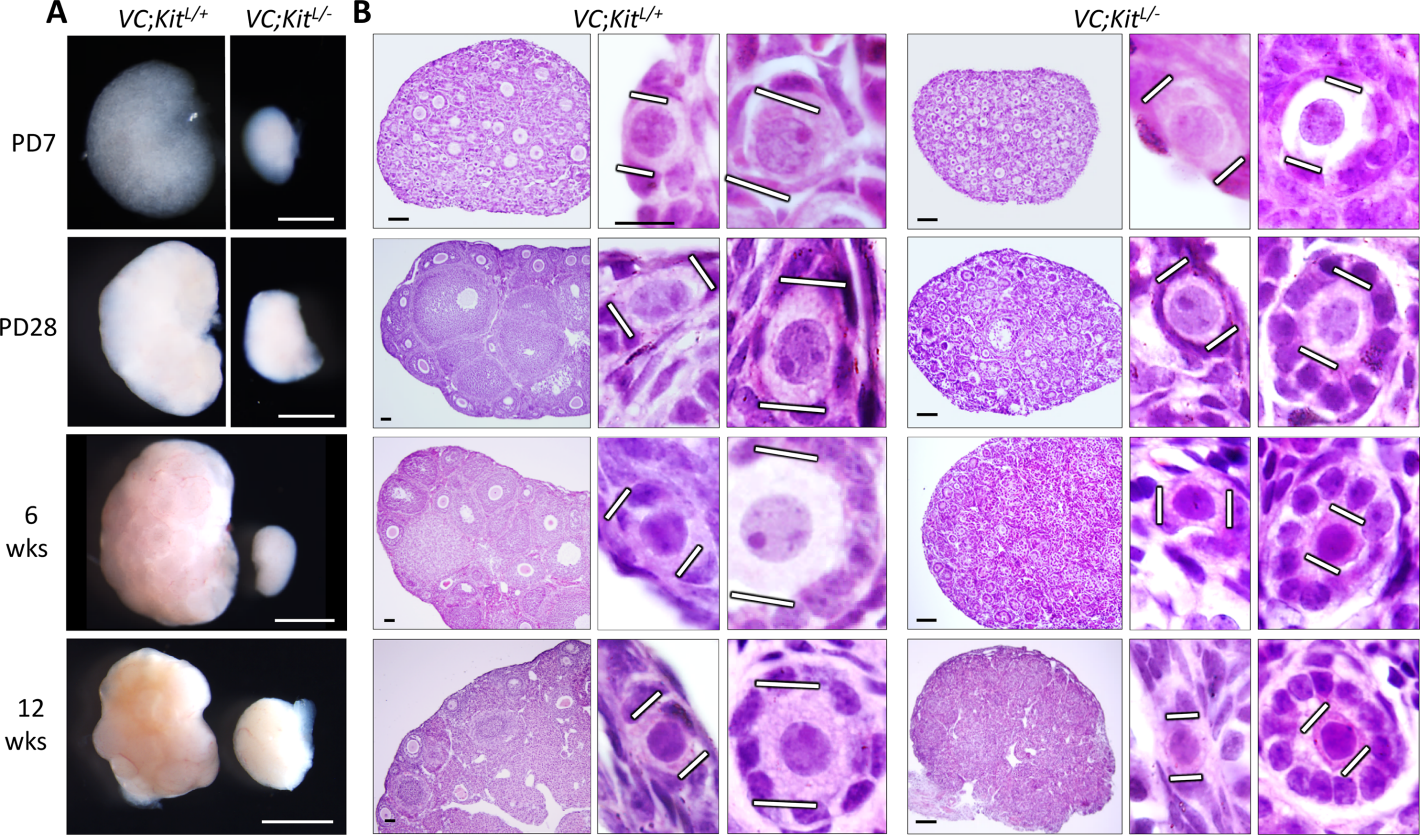
Primordial follicle arrest phenotype following Kit inactivation in oocytes. (A) *VC*; *Kit*^*L/-*^ ovaries were minute relative to *VC; Kit*^*L*^*/+* sibling controls at all timepoints analyzed; scale bar = 1 mm. (B) Histological analyses (H&E-stained sections) revealed abundant primordial follicles with a complete failure of oocyte growth and absence of advanced follicles in *VC*; *Kit*^*L/-*^ ovaries. White lines demarcate oocyte diameters. Scale bars = 50 μm (large panels) or 10 μm (small panels). Pictures are representative of ≥4 ovaries from ≥2 different animals per timepoint.

At PD7, *VC*; *Kit*^*L/-*^ovaries were minute, and their small size persisted at all mouse ages analyzed, up to 12 weeks of age (Fig 5A). Histological analyses revealed a striking and complete failure of oocyte reawakening. Follicles remained small and no growing oocytes were present, although granulosa cells became cuboidal. In these follicles, oocytes typically assumed an eccentric location (Figs 5B and S6A). Electron microscopy (EM) confirmed the viability of these oocytes and their lack of physical growth. Granulosa cells exhibited some mitotic activity in the mutant follicles per Ki67 immunohistochemistry (whereas normal primordial follicles are mitotically inactive) (S6D Fig), consistent with increased granulosa cell numbers in the aberrant follicles of *VC*; *Kit*^*L/-*^ovaries. Interestingly, EM also revealed abundant lipid droplets in the granulosa cells, which could contribute to their change in shape (S6B Fig). The significance of these lipid droplets is uncertain. One interpretation is that granulosa cells “sense” the lack of oocyte growth (due to known, if poorly understood, bidirectional oocyte/granulosa cell communication), and respond in some manner to promote reawakening [5, 40]. Alternatively, an abnormal hormonal milieu associated with ovarian failure could also indirectly contribute to the observed changes in granulosa cells. In any case, ZP1, which is expressed in growing oocytes, was not expressed in any *VC*; *Kit*^*L/-*^ oocytes, consistent with a constitutional inability to reawaken/initiate growth despite the granulosa cell changes.

**Fig 6:**
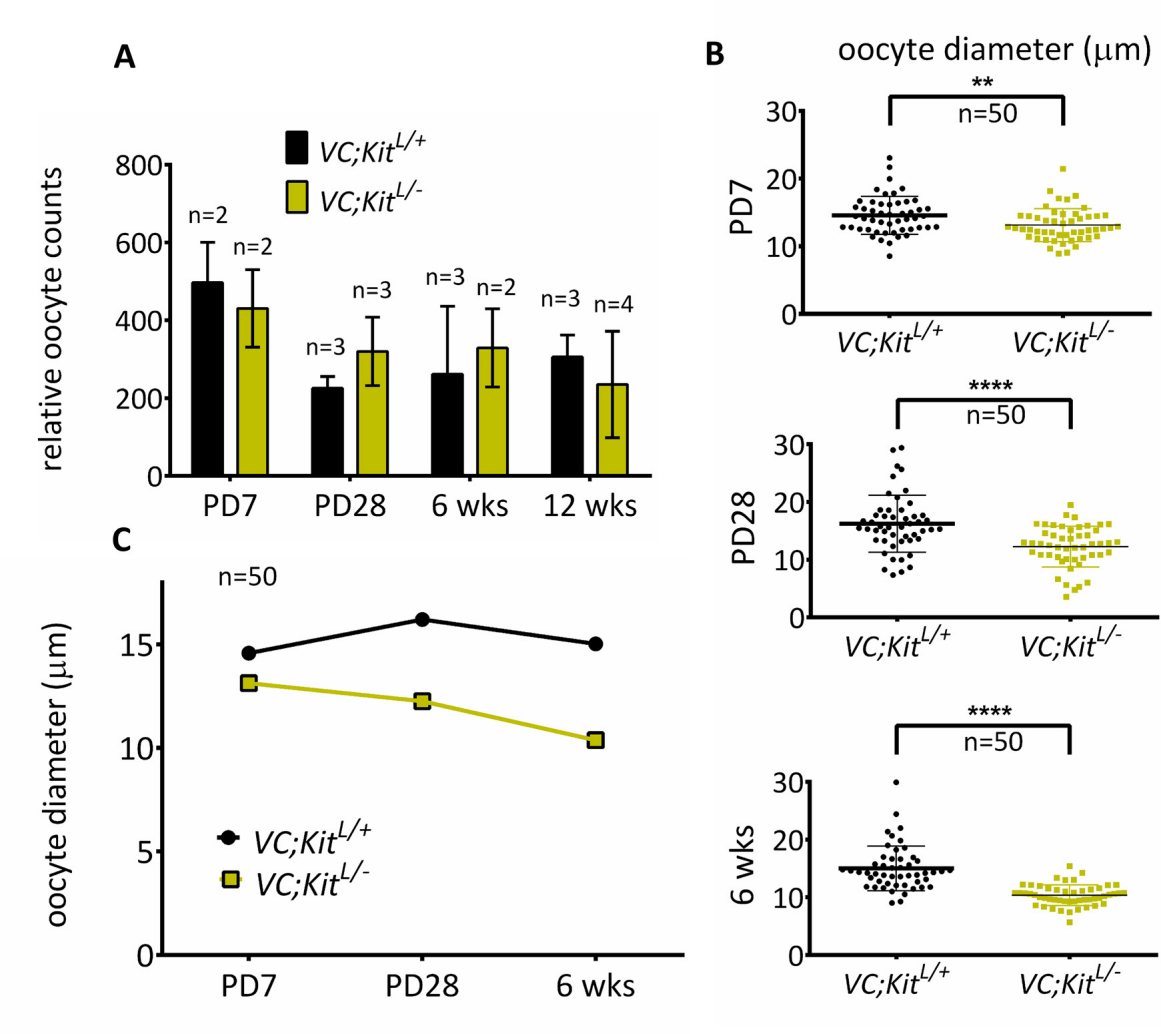
Quantitative analyses of oocyte phenotypes following Kit inactivation in oocytes. (A) Relative oocyte numbers at PD7 to 12 wks; n = 3 animals per genotype per timepoint except PD7 and 6 weeks experimental (n = 2 animals, total of 4 ovaries analyzed per genotype), error bars = S.E.M. Note that there is no significant oocyte loss up to 12 weeks (p = 0.4475 at 12 weeks, unpaired student t-test). (B) Oocyte diameters (micrometers) of early (primordial/primary) oocytes; **p<0.01, ****p<0.0001; unpaired student t test; n = 50 per genotype. **(C)** Average oocyte diameters, n = 50 oocytes per genotype per timepoint.

Oocyte numbers were unaltered in *VC*; *Kit*^*L/-*^ovaries at PD7, demonstrating that the minute ovaries were due to a complete lack of oocyte growth, and not a diminished initial endowment of primordial follicles (Fig 6A). Measurements of oocyte diameters confirmed the lack of oocyte growth (Fig 6B and 6C). These quantitative and morphometric data thus revealed a complete failure of oocyte reawakening in *Kit*-deficient oocytes. Somewhat unexpectedly given prior studies implicating Kit in germ cell and primordial oocyte survival, *VC*; *Kit*^*L/-*^ oocytes did not undergo rapid apoptosis [28, 41]. To the contrary, *VC*; *Kit*^*L/-*^ primary/primordial oocyte counts showed only a minor (statistically not significant) decrease even at 12 weeks of age, consistent with a remarkably specific role for Kit in oocyte reawakening (Fig 6A). However, by 6 months of age, the ovaries were entirely depleted of follicles and oocytes and contained only luteinized stroma (S7A–S7C Fig), demonstrating that Kit is required for the long-term maintenance of oocytes, in keeping with prior studies implicating Kit as an oocyte survival factor [28, 42].

**Fig 7:**
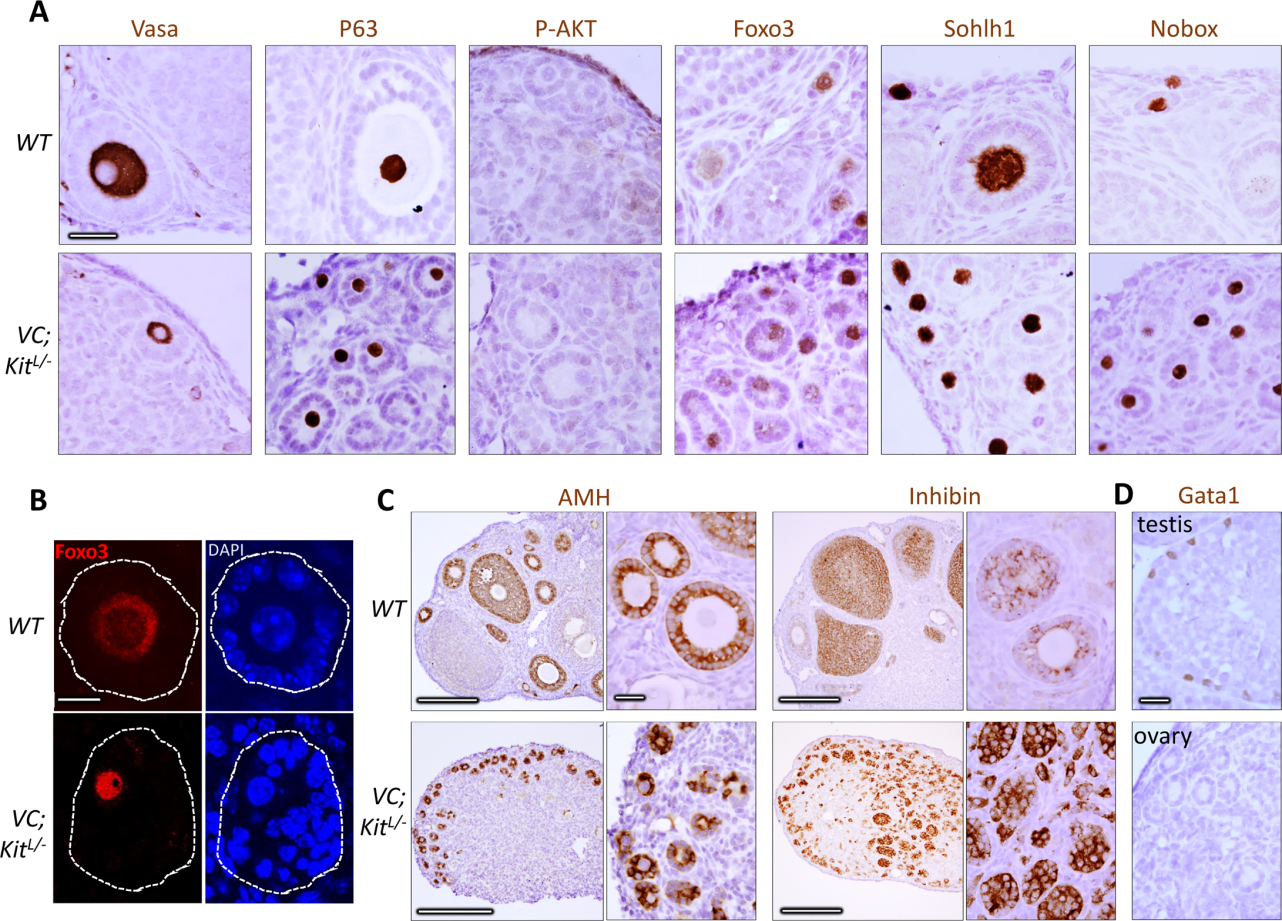
Marker studies of Kit-deficient oocytes are consistent with specific defect in oocyte reawakening via Foxo3. (A) Immunohistochemistry for markers as shown at 6 weeks of age; slides counterstained with hematoxylin. Note absence of P-AKT (in contrast to D818V mutant) and constitutively nuclear localization of Foxo3. Scale bars = 25 μm; all panels at same magnification. (B) Immunofluorescence detection of Foxo3 protein; slides counterstained with DAPI. Foxo3 undergoes nuclear to cytoplasmic export in wild-type primary follicles but is constitutively nuclear in the mutant. Scale bar = 10 μm, same for all panels. (C) Analysis of granulosa cells in wild-type and VC; Kit^L/-^ ovaries. Immunohistochemistry for markers as shown at 6 wks of age; slides counterstained with hematoxylin. Scale bars = 200 μm in large panels, or 25 μm in small panels; all small panels at same magnification. (D) Absence of Gata1-positive cells in aberrant follicles in VC; Kit^L/-^ ovaries. Wild-type testis is shown as a positive control (Gata1 is expressed in Sertoli cells). Scale bar = 25 μm; both panels at same magnification.

At birth, oocytes are syncytial and interconnected by intercellular bridges, which are broken down by PD3 to give rise to individualized primordial follicles. Follicle individualization (also known as assembly) occurred normally in *VC*; *Kit*^*L/-*^ovaries (e.g., no follicles contained more than one oocyte), demonstrating that Kit is not essential for individualization despite its abundant expression within oocytes at PD1-3 when individualization takes place (Fig 5B). Additional marker studies at 6 weeks of age showed that all oocytes retained germline identity, with normal expression of primordial oocyte markers such as Vasa, p63 (an oocyte survival factor), Foxo3, Sohlh1, and Nobox (Fig 7A). Granulosa cells continued to express inhibin and AMH (markers of female gonadal somatic cell differentiation) but did not express the Sertoli cell marker Gata-1 (Fig 7C and 7D), evidence against a sex-reversal phenotype, a possibility entertained because of the morphologic resemblance of the aberrant follicles—particularly those with eccentric oocytes—to primitive male sex cords. These results are consistent with a specific role of Kit in oocyte reawakening. Additionally, Foxo3 was constitutively nuclear and P-AKT was undetectable in *Kit*-deficient oocytes (Fig 7A and 7B) further supporting a critical role of the Kit signaling pathway in regulating oocyte awakening via P-AKT/Foxo3.

## Discussion

Primary ovarian insufficiency (POI), also known as premature ovarian failure, is a form of hypergonadotropic hypogonadic ovarian failure that causes early menopause and infertility in 1% of women before the age of 40, in addition to other important health consequences due to estrogen deficiency [43]. POI is associated with the accelerated depletion of primordial follicles, arguing that abnormalities in primordial follicle maintenance are the unifying pathophysiologic basis of POI. However, the identification and further study of key factors regulating primordial follicle maintenance and reawakening has proven difficult with human subjects; for example, ovaries are not biopsied in the clinical workup of POI, limiting available human ovarian tissue for direct analyses. Genome-wide studies have implicated several loci, but further and more detailed genome-wide investigations are needed to more fully define the genetic basis of POI [44–47]. Numerous factors have been proposed as regulators of reawakening, such as the AMH type 2 receptor (AMHR2), a member of the transforming growth factor β superfamily of growth and differentiation factors [48]. *AMHR2* female mice are fertile with no overt defects in follicle maturation, however, arguing against an essential role in reawakening [49]. Similarly, while α-Müllerian hormone (AMH) has also been proposed as a regulator of oocyte utilization and reawakening, AMH-null females are fertile, suggesting that AMH plays at most a secondary role in regulating reawakening. Other factors must therefore participate in this process [50].

Kit has also been proposed as a candidate factor regulating reawakening. Kit and Kit ligand (KL, also known as SCF) serve diverse functions in the germ cell lineage, particularly in primordial germ cell migration/survival, primordial follicle assembly [51], and spermatogenesis. KL is produced by granulosa cells in primordial follicles [32] and Kit is highly expressed by primordial oocytes. Thus, patterns of Kit and KL expression within the ovary make them plausible candidates as factors regulating reawakening. *In vitro* studies conducted with explanted ovaries treated with KL or Kit inhibitors have also been interpreted as supportive of this hypothesis [52, 53]. On the other hand, the observed effects have been relatively small in magnitude and more importantly, these *in vitro*, non-genetic approaches suffer from significant potential limitations stemming from the unique biological properties of reawakening. Reawakening normally occurs at a gradual and measured pace throughout life, such that only a very small percentage of follicles are awakening at any time. Thus, available *in vitro* methods with explanted neonatal ovaries (which can be maintained for only one to two weeks) do not provide a completely adequate timescale for definitive studies including the identification of novel factors regulating reawakening.

Phenotypic analysis of *Kit*^*Y719F*^ homozygous female mice have provided intriguing genetic evidence implicating Kit in reawakening [28]. Kit activates PI3K through a direct interaction with an SH2 domain on the p85 regulatory subunit of PI3K. This interaction is dependent on Kit tyrosine residue 719, which undergoes autophosphorylation following ligand binding. Mutation of this tyrosine residue prevents binding of Kit to p85, and thus abrogates Kit signaling via PI3K [54]. Mice engineered with this *Kit*^*Y719F*^ mutation (via a “knockin” approach) have permitted genetic dissection of the contribution of PI3K signaling to diverse Kit-dependent biological processes [55, 56]. Homozygous *Kit*^*Y719F*^ males are sterile with severe defects in spermatogenesis. *Kit*^*Y719F*^ females, however, are fertile at 16 weeks of age, suggesting that Kit might not play an essential role in reawakening (although severe defects in early follicle maturation including an arrest at the primary/secondary follicle stage were documented) [28]. Subsequent analyses of this allele have been interpreted as supportive of a role of Kit in reawakening, rationalizing its use in studies to genetically dissect reawakening. For example, genetic inactivation of *Tsc1* within primordial follicle granulosa cells leads to global reawakening via Foxo3 cytoplasmic relocalization, and this was found to occur through enhancement of Kit ligand production in primordial follicle granulosa cells. *Kit*^*Y719F*^ homozygosity suppressed this *Tsc1*-null reawakening phenotype, arguing that the observed increase in Kit ligand was responsible for reawakening [57].

Foxo3 relocalization to the cytoplasm occurs after the primordial-primary transition, and abnormal Foxo3 relocalization has been documented in mutants undergoing global reawakening [12, 13, 16, 57]. However, for reasons that are not well understood, Foxo3 relocalization as visualized by IHC/IF does not anticipate (precede) reawakening; i.e., early primary follicles have nuclear Foxo3 [13]. It is possible that Foxo3 protein can be functionally inactivated in the oocyte but that the protein remains detectable in the nucleus for some time. Thus, in practice, the most useful, sensitive, and earliest indicators of reawakening (i.e., for scoring mutant phenotypes) remain morphologic. As emphasized in this study, oocyte diameter is the earliest and most sensitive endpoint for reawakening. In diverse investigations of mutants with global reawakening (e.g., *Pten* and *Foxo3* mutants, also in the *Kit*^*D818V*^ mutant described here), granulosa cells remain flattened and unilayered in many follicles with massively enlarged oocytes that have clearly undergone reawakening; indeed, such oocytes can continue to grow unabated for several weeks despite the persistence of “primordial follicle-like” granulosa cell morphology [10, 13, 16, 19, 58]. Furthermore, we have here documented in *VC;Kit*^*L/-*^ ovaries a transition in granulosa cell morphology to a cuboidal shape and mitotic activity resulting in a few layers even in follicles where oocytes were constitutionally unable (due to Kit inactivation) to grow/reawaken. Thus, while granulosa cells normally undergo significant changes in morphology and arrangement during reawakening, these do not always correlate with oocyte status in individual follicles and are not as useful as morphological indicators in mutants with strong reawakening phenotypes. Thus, we stress that abnormal oocyte diameters should be considered the *sine qua non* for scoring oocyte reawakening phenotypes and that careful measurements of oocyte diameters (e.g., in tissue sections) should represent the gold standard in such analyses until earlier molecular markers of reawakening are identified.

That the *Kit*^*Y719F*^ mutation did not lead to a complete oocyte dormancy phenotype, as documented in earlier studies (females were fertile up to at least 16 weeks of age) can be explained by the hypomorphic nature of this mutation [28]. For example, residual Kit signalling via PI3K or other surrogate signalling pathways could feed back to PI3K, resulting in some PI3K tonic activity. Finally, some hypomorphic alleles of KL (a.k.a. *Steel*^*panda*^) also accumulate follicles with cuboidal granulosa cells (albeit with growth-arrested oocytes), consistent with a role of KL in reawakening, although these ovaries contain very few follicles, obscuring interpretation of phenotypes [28, 59]. The role of KL in reawakening should be further explored in future studies; i.e., through conditional genetic analysis of KL in granulosa cells. KL appears to be constitutively expressed in granulosa cells in primordial follicles [60], and although KL is elevated in the *Tsc1* reawakening mutant, the nature of the signals and inter/intra-follicular communication triggering reawakening via Kit-PI3K-Foxo3 in individual primordial follicles remains uncertain. More recently, conditional deficiency of the Lim homeodomain protein Lhx8 was found to promote global reawakening, with a synergistic effect of Lhx8 and Pten on Foxo3 nucleocytoplasmic translocation. These effects were mediated by Lin28a, an RNA binding protein and regulator of the let-7 microRNAs, a finding of particular interest given studies implicating the Lin28/let-7 axis in the control of PI3K-mTOR signalling [16, 61]. Clearly, more work is needed to fully dissect the interactions of the diverse members of the PI3K and mTOR pathways and how these function together to trigger reawakening of individual primordial follicles.

One notable aspect of this study is that we have now documented global oocyte reawakening and dormancy phenotypes with our *Kit*^*D818V*^ and *Kit*^*L*^ alleles, respectively. Such complementary and contrasting phenotypes with alleles that have opposite effects on Kit activity (i.e., gain- vs. complete loss-of-function alleles) represent compelling genetic evidence incriminating Kit as the critical receptor upstream of PI3K-Foxo3 in the control of reawakening. The phenotype we describe for the oocyte conditional *Kit* knockout represents the first report of a pure global dormancy mutant; i.e., where female sterility was associated with minute ovaries containing a numerically normal complement of primordial follicles but where a complete failure of oocyte reawakening left oocytes incapable of growth/reawakening. Conversely, Kit serves essential roles in other aspects of follicle formation, development, and survival [3, 28, 51].

Our genetic studies do not exclude a contribution from other cell surface receptors in reawakening, but help establish Kit as the principal upstream factor regulating reawakening, and also demonstrate a remarkably specific role for Kit in reawakening. It is surprising that Kit played such a modest role in oocyte survival, with normal oocyte counts up to 12 weeks of age, a result suggesting that other factors regulate oocyte survival and prevent apoptosis [62]. Oocyte conditional ablation (*Vasa-Cre*) of other genes encoding cell surface receptors acting through PI3K and known to be expressed on the oocyte, such as *Igf1r*, *Insr*, *Ret*, *Pdgfr* had no effect on fertility or follicle maturation (unpublished data), further stressing the uniqueness of the *Kit* reawakening phenotypes.

POI is a form of hypergonadotropic hypogonadism that causes infertility in 1% of women before the age of 40 and has important health consequences. POI is due to accelerated depletion of primordial follicles [63–66], arguing that abnormalities in primordial follicle maintenance are the unifying pathophysiologic basis of POI. However, the molecular mechanisms that cause follicle depletion in POI are poorly understood. Elucidating these mechanisms is needed to develop better treatment strategies and assays predictive of POI. Our results should help focus further investigations on Kit and, by extension, Kit ligand, as keystone regulators of primordial oocyte maintenance and hence, female reproductive aging. Pharmacologic manipulation of this pathway may someday prove useful in fertility preservation by increasing the pool of actively growing follicles [14]. This is further underscored by the fact that PI3K-Foxo3 signaling regulates the egg supply throughout life [28]. For example, transient treatment of mouse or human oocytes with either a Pten inhibitor or a PI3K activating peptide results in activation of dormant primordial follicles. Pharmacologic control of this pathway either at the level of PI3K-AKT or Kit-KL may thus prove useful in fertility preservation by increasing the pool of growing follicles [14, 15, 67].

## Materials and Methods

### Ethics statement

All live animal experiments followed guidelines by the UTSW Animal Care and Use Committee who also approved the experiments performed (2015–101272).

### Mouse strains and genotyping

Mice were housed in a pathogen-free animal facility in microisolator cages and fed ad libitum on standard chow under standard lighting conditions. A detailed description of the targeting strategy, assembly, primer sequences, and PCR conditions for generating the *Kit*^*D818V(L)*^ and *Kit*^*L*^ alleles are provided in S1 Text.

Mice were genotyped from tail DNA by PCR with Promega GoTaq in 1.6 mM MgCl_2_. Genotyping primers for the *KitD818V* allele were as follows: (a1) 5’-ATTAGAGCCCCGATCCTGTG-3’ and (b1) 5’-GCAACAGCCATTCATTTCAGC-3’ (see S1 Fig for positions of a1/b1 primers), under the following cycling conditions: 95° x 2 min; 94° x 30 sec, 60° x 30 sec, 72° x 30 sec (35 cycles); 72° x 7 min. The product sizes are 219 bp for the floxed allele *Kit*^*D818V(L)*^ and 171 bp for the wild type *Kit* allele. Genotyping primers for *Kit*^*L*^ allele are as follows: (a1) 5’-AGTTCTGAAGAGACTGTCAAGGT-3’ and (b2) 5’-ACACCCCATTTCCTTATTTTTGCT-3’(see S3 Fig for positions of a1/b1 primers), under the following cycling conditions: 95° x 2 min; 94° x 30 sec, 60° x 30 sec, 72° x 30 sec (35 cycles); 72° x 7 min. The product sizes are 174 bp for the floxed *Kit*^*L*^ allele and 126 bp for the wild type *Kit* allele.

### RNA isolation and Sanger sequencing

Total RNA was prepared from ovaries with the Tripure isolation reagent (Roche #93876820) per the manufacturer’s instructions. To validate the *Kit*^*D818V(L)*^ allele, exon 17 was amplified by one step RT-PCR (Qiagen #210210) using the primers: 5’-AGATTTGGCAGCCAGGAATA-3’ (forward) and 5’-ATTTCCTTTGACCACGTAATTC-3’ (reverse). For validation experiments of the *Kit*^*-*^ allele, cDNA was synthesized with M-MuLV Reverse Transcriptase (New England BioLabs #28025–013). Kit cDNA sequences, spanning exons 14 to 18 were amplified by Phusion High-Fidelity DNA Polymerase (New England BioLabs #M0530S). The forward primer (close to exon 14) was 5’-GAGAAGGAAGCGTGACTC-3’; the reverse primer (close to exon 18) was 5’-AGGAGAAGAGCTCCCAGA-3’. PCR conditions were: 98°C x 3 min; 34 cycles of 98°C x 60 sec, 60°C x 60 sec, 72°C x 120 sec; then 72°C x 5 min. RT-PCR products were analyzed by gel electrophoresis and bands purified with the QIAquick gel extraction kit (Qiagen #28704). The *Kit*^*D818V*^ mutation or exon 17 deletion were confirmed by Sanger sequencing (UTSW sequencing core).

### Tissue processing, immunohistochemistry (IHC), immunofluorescence (IF), and tissue analyses

Ovaries were fixed in 10% formalin overnight, embedded in paraffin and serially sectioned (5 μm). Every fifth section was H&E stained and analyzed. For *VC*; *Kit*^*D818V(L)*^/+ and control ovaries, the middle section of the series was used for relative follicle counts and diameter measurements as described in the text. For *VC*; *Kit*^*L/-*^ and control ovaries, all the primordial and primary oocytes were counted in every fifth section. The longest diameter of 50 oocytes for which nuclei were in the plane of section was determined with ImageJ. For IHC, sections were rehydrated in EtOH series after deparaffinization in xylene. Antigen retrieval was performed in parboiling 10 mM sodium citrate (pH 6.0) x 20 min, cooled at RT, followed by peroxidase blocking (3% H_2_0_2_) and blocking in 0.5–1% BSA in PBS. Antibodies and titers for IHC were: Kit 1:750 (Cell Signaling #3074S); Vasa 1:200 (Abcam #27591); P-AKT 1:75 (Cell Signalling #S473); Sall4 1:1000 (Abcam #57577); Foxo3 1:50 (Santa Cruz #sc-11351); AMH 1:500 (Serotec #MCA2246); P63 1:500 (Thermo Fisher #MS-1081); Inhibin (Biorad #MCA951ST); Gata-1 (Santa Cruz #sc-265); Zp1 (Santa Cruz #sc-23708); Gdf9 (Santa Cruz #sc-12244); Ki67 (Abcam #15580); Sohlh1 and Nobox antibodies were kindly provided by Dr. Aleksandar Rajkovic (Magee-Womens Research Institute). ImmPRESS (Vector Laboratories) was used for detection. For immunofluorescence (IF), sections were rehydrated in an EtOH series after deparaffinization in xylene. Antigen retrieval was performed in parboiling 10 mM sodium citrate (pH 6.0) x 20 min, cooled at RT, followed by PBS washes, 10 minutes of autofluorescence blocking (100 nm Tris Glycine), and blocking in 2.5% BSA+5% goat serum (Vector Laboratories) in PBS. Antibodies and titers for IF were: Foxo3 1:50; Kit 1:100. Fluorophore conjugated secondary mouse (Alexa Fluor 555) or rabbit (Alexa Fluor 488) IgGs at 1:500 (Invitrogen #A21429 and #A11029) were used for detection. Zeiss LSM 510 confocal microscopy was used for fluorescence imaging.

### FSH and LH analyses

Serum FSH/LH levels were measured by the Ligand Assay Core at the University of Virginia. Serum was diluted 1:10 prior to analysis.

### Western blot

Ovaries (two/genotype) were homogenized in RIPA buffer supplemented with Complete Proteinase inhibitor cocktail (Roche) and Phosphatase Inhibitor cocktail 2 (Sigma). Following homogenization, extracts were centrifuged at 10000 rpm for 7 min. Supernatants were collected, mixed with 4x Laemmli Sample Buffer (BioRad) and boiled for 10 min. Equal amounts were loaded in a 10% SDS PAGE and run at 100V. Gel was transferred to Immobilon P membrane (Millipore). The membrane was blotted with 5% dry milk in TBS-T (BB), and probed with 1:1000 dilution of Kit antibody in BB overnight at 4°C (Cell Signaling, #3074). The membrane was stripped with Restore Stripping Buffer (Thermo Scientific), blotted and probed with 1:5000 α-tubulin in BB for 1hr at room temperature (Sigma, T9026). Blots were developed with SuperSignal West Dura Extended Duration Substrate (Thermo Scientific) and digital images acquired with a ChemiDoc system (BioRad).

### Toluidine blue staining and electron microscopy

Two ovaries per genotype were fixed, embedded in Embed 812 resin (Electron Microscopy Sciences) and prepared for negative staining as described [9]. Images were acquired using a FEI Tecnai G2 Spirit electron microscope. Thin sections were also stained with Toluidine blue and analyzed with light microscope.

### Statistical analysis

P values and means +/- S.E.M. were calculated by two-tailed unpaired Student’s t test with GraphPad Prism 6.

## Supporting Information

S1 Fig Generation of Kit^D818V(L)^ allele. (A) Maps of Kit locus (exons 14–21) and targeting construct. The modified exon 17 is shown in red and the D818V point mutation is indicated with an asterisk. Unmodified exons are represented in blue, the 3’ cDNA cassette encoding exons 17–21 and polyA sequence in green. Genotyping primers are shown by small arrows (a1 and b1). (B) Normal pigmentation in Kit^D818V(L)^/+ mice at 4 weeks of age. (C) PCR genotyping of wildtype, Kit^D818V(L)^/+ heterozygous, and Kit^D818V(L)^ homozygous mice from tail DNA. The 219 bp band corresponds to D818V(L), while the 171 bp product corresponds to the wild-type allele. (D) Total RNA was isolated from PD7 wild-type and VC; Kit^D818V(L)^/+ ovaries and analyzed by Sanger sequencing following RT-PCR. The expected two base-pair substitution resulting in an Asp➔Val substitution was observed.
(TIF)

S2 Fig Analysis of Kit expression in *VC; Kit*^*D818V(L)/+*^ ovaries. (A) Kit immunohistochemistry at PD14. White asterisks indicate normal primordial follicles; white arrows point to growing follicles. Scale bar = 33 μm; both large panels are at same magnification. (B) Kit and Foxo3 double-labeling at PD14. Foxo3 is nuclear and cytoplasmic in control oocytes but exclusively cytoplasmic in experimental oocytes. Scale bar = 33 μm; all panels are at the same magnification. (C) Kit immunohistochemistry at PD14; slides are counterstained with hematoxylin. Scale bar = 25 μm; both panels at same magnification. (D) Western analysis of PD7 ovaries.
(TIF)

S3 Fig Reawakened oocytes differentiate normally in *VC; Kit*^*D818V(L)/+*^ ovaries (PD14). Tissue sections stained with periodic acid-Schiff (PAS) or analyzed by immunohistochemistry and counterstained with hematoxylin. PAS and Zp1 staining indicate development of zona pellucida. Granulosa cells express the differentiation marker, inhibin, and oocytes express Gdf9, Sohlh1 and Nobox. Insets show higher magnification. Scale bars = 25 μm; top and bottom panels at same magnification.
(TIF)

S4 Fig Loss of the primordial follicle pool due to oocyte reawakening. (A) Oocyte reawakening phenotype at PD7 and PD28. Immunohistochemistry for markers as shown; slides counterstained with hematoxylin. White arrows indicate follicles with atretic (Sall4-negative) oocytes; black arrows indicate AMH-negative granulosa cells. Scale bar = 25 μm; all panels at same magnification. (B) Serum FSH and LH levels of VC; Kit^D818V(L)^/+ and control females at five months of age. *p<0.05, **p<0.01; unpaired student t test; n = 3 animals per genotype. (C) Toluidine-blue stained sections of plastic-embedded ovaries from experimental and sibling controls at 6 weeks of age; scale bar = 10 μm, both panels at same magnification. The follicle shown on the right does not contain a viable oocyte.
(TIF)

S5 Fig Generation of *Kit*^*L*^ *allele* (exon 17 floxed). (A) Maps of *Kit* locus (exons 14–21) and targeting construct. Genotyping primers are indicated by small arrows (a1 and b1). (B) PCR genotyping of wild-type, *Kit*^*L/+*^ and *Kit*^*L/L*^ mouse tails. The 174 bp product corresponds to the *Kit*^*L*^ allele; the 126 bp product corresponds to the wild-type allele. (C) cDNA expression analysis of mutant allele. cDNA was synthesized from PD3 wild-type and *VC; Kit*^*L/-*^ ovaries and a region spanning from exon 14 to exon 18 was amplified. The 417 bp (lower) product corresponds to the Cre-mediated exon 17 deletion, while the 545 bp (upper) product corresponds to the wild-type *Kit* allele. (D) Sanger sequencing of 417 bp cDNA product from *VC*; *Kit*^*L/-*^ ovaries confirms precise exon 17 deletion. (E) Mice hemizygous for *Kit* at 4 weeks of age (but not mice harboring floxed allele, left) show hypopigmentation and midline pigmentation defects.
(TIF)

S6 Fig Ultrastructural analysis and absence of zona pellucida in *VC*; *Kit* ^*L/-*^ follicles. (A) Toluidine-blue stained sections highlight presence of viable but small oocytes that were often eccentrically located within follicles in *VC; Kit* ^*L/-*^ ovaries. Note lipid droplets in granulosa cells shown by red arrows. Dashed lines demarcate oocyte diameters (30 μm for control, 21 μm for mutant). Scale bars = 10 μm. (B) Transmission electron microscopy of control (top) and *VC*; *Kit* ^*L/-*^ ovaries. Note presence of zona pellucida (black asterisk) surrounding a large oocyte in control (left panel). Right panels show high-magnification views of single granulosa cells (indicated by black boxes). In the mutant, note the small, eccentrically-located oocyte and complete absence of a zona pellucida. Higher magnification views of granulosa cells confirm lipid droplets in the mutant (red asterisk). Scale bars = 10 μm for follicle images, 2 μm for granulosa cells. (C) Zp1 immunohistochemistry at 6 weeks of age; slides counterstained with hematoxylin. Red arrows point to small follicles; Zp1 is readily detected in control oocytes. Scale bars = 25 μm, same for both panels. (D) Ki67 immunohistochemistry at 4 weeks of age; slides counterstained with hematoxylin. Red arrows indicate granulosa cells. Scale bars = 25 μm, same for both panels.
(TIF)

S7 Fig *VC; Kit*^*L/-*^ oocytes die by 6 months of age. (A) Gross pictures of ovaries from control and *VC; Kit*^*L/-*^ females. Scale bar = 1 mm. (B) Histological analyses (H&E-stained sections) reveal complete absence of follicles in *VC; Kit*^*L/-*^ females. Scale bars = 25 μm for all panels. (C) Loss of oocytes by 6 months of age analyzed Vasa immunohistochemistry. Scale bars = 25 μm, same for all panels.
(TIF)

S1 Text Supplementary Materials and Methods. (PDF)
