# Supplementary figures and images for: Control of Oocyte Reawakening by Kit
Source: PLoS Genet. 2016 Aug 8;12(8):e1006215. doi: 10.1371/journal.pgen.1006215 (PMC4976968; doi:10.1371/journal.pgen.1006215)

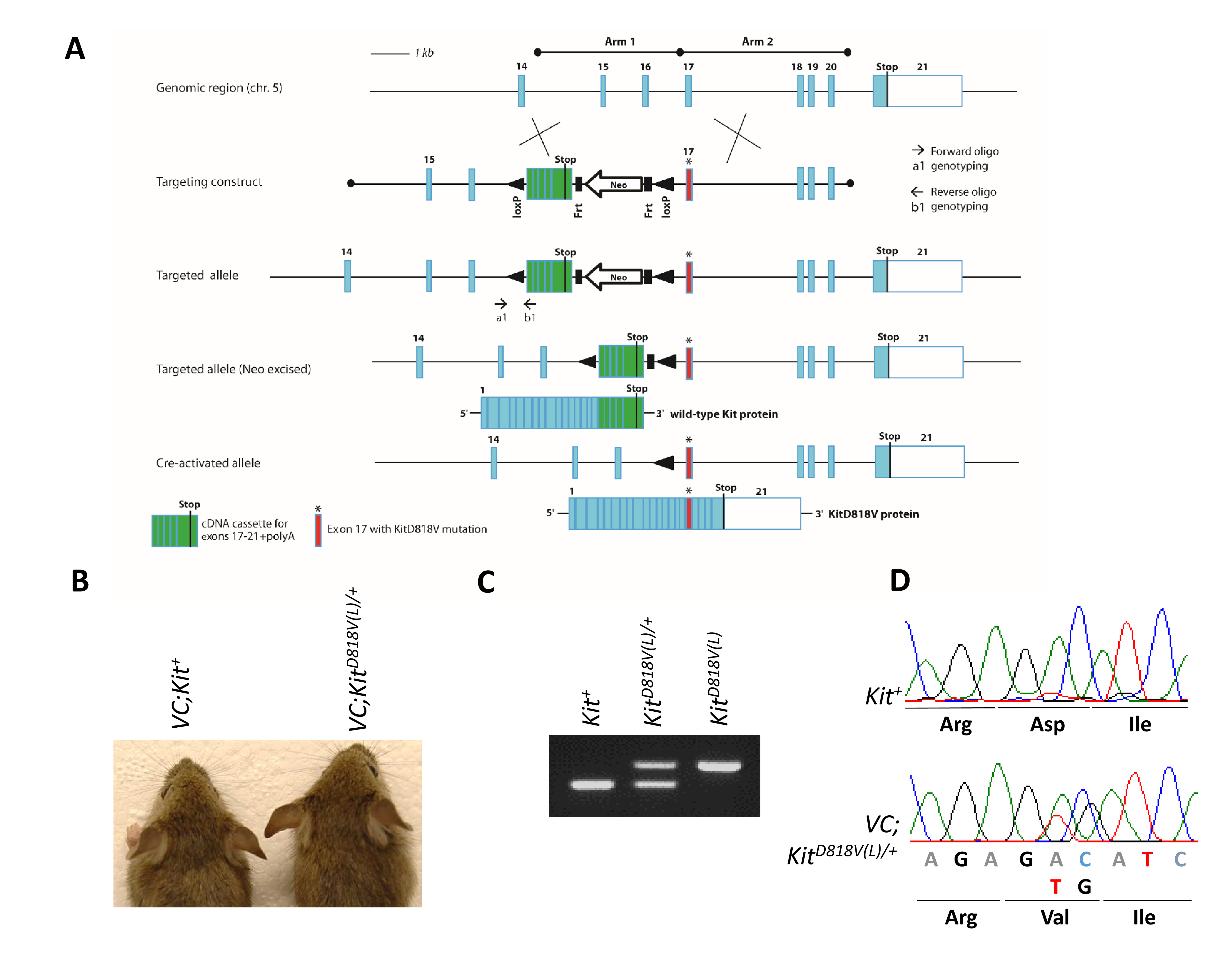

Supplement: S1 Fig — (A) Maps of Kit locus (exons 14–21) and targeting construct. The modified exon 17 is shown in red and the D818V point mutation is indicated with an asterisk. Unmodified exons are represented in blue, the 3’ cDNA cassette encoding exons 17–21 and polyA sequence in green. Genotyping primers are shown by small arrows (a1 and b1). (B) Normal pigmentation in KitD818V(L)/+ mice at 4 weeks of age. (C) PCR genotyping of wildtype, KitD818V(L)/+ heterozygous, and KitD818V(L) homozygous mice from tail DNA. The 219 bp band corresponds to D818V(L), while the 171 bp product corresponds to the wild-type allele. (D) Total RNA was isolated from PD7 wild-type and VC; KitD818V(L)/+ ovaries and analyzed by Sanger sequencing following RT-PCR. The expected two base-pair substitution resulting in an Asp➔Val substitution was observed. (TIF) [file pgen.1006215.s001.tif]

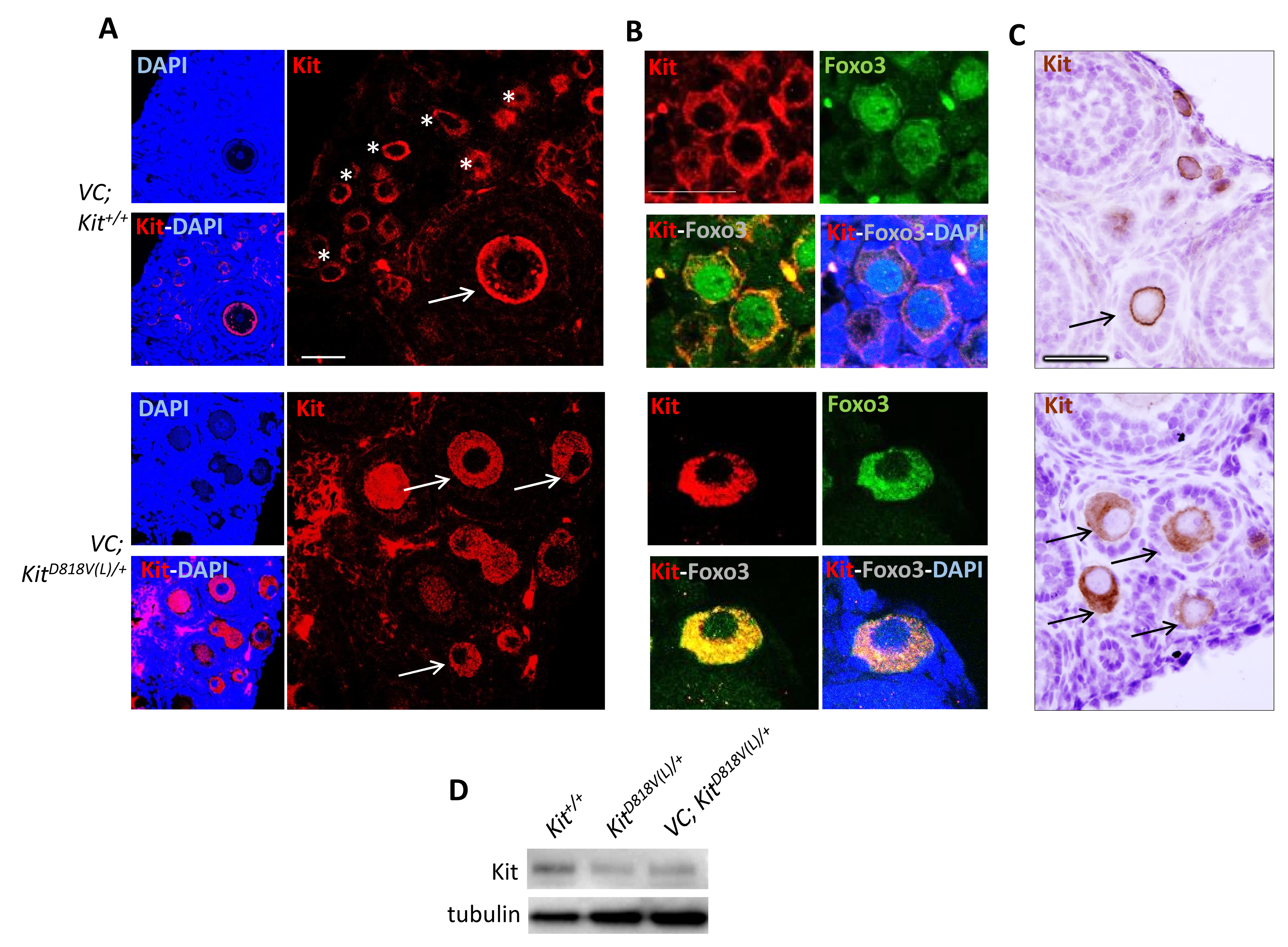

Supplement: S2 Fig — (A) Kit immunohistochemistry at PD14. White asterisks indicate normal primordial follicles; white arrows point to growing follicles. Scale bar = 33 μm; both large panels are at same magnification. (B) Kit and Foxo3 double-labeling at PD14. Foxo3 is nuclear and cytoplasmic in control oocytes but exclusively cytoplasmic in experimental oocytes. Scale bar = 33 μm; all panels are at the same magnification. (C) Kit immunohistochemistry at PD14; slides are counterstained with hematoxylin. Scale bar = 25 μm; both panels at same magnification. (D) Western analysis of PD7 ovaries. (TIF) [file pgen.1006215.s002.tif]

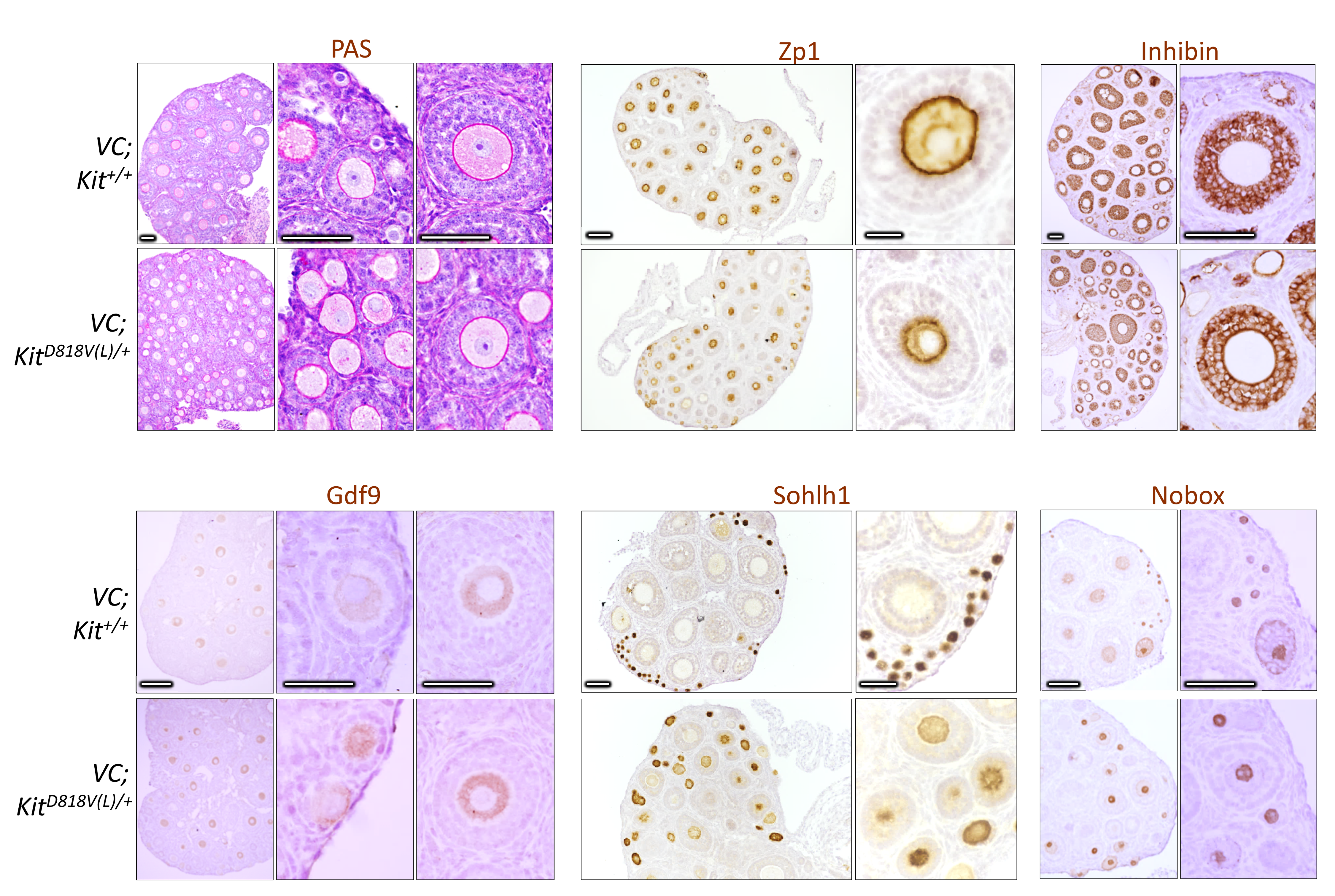

Supplement: S3 Fig — Tissue sections stained with periodic acid-Schiff (PAS) or analyzed by immunohistochemistry and counterstained with hematoxylin. PAS and Zp1 staining indicate development of zona pellucida. Granulosa cells express the differentiation marker, inhibin, and oocytes express Gdf9, Sohlh1 and Nobox. Insets show higher magnification. Scale bars = 25 μm; top and bottom panels at same magnification. (TIF) [file pgen.1006215.s003.tif]

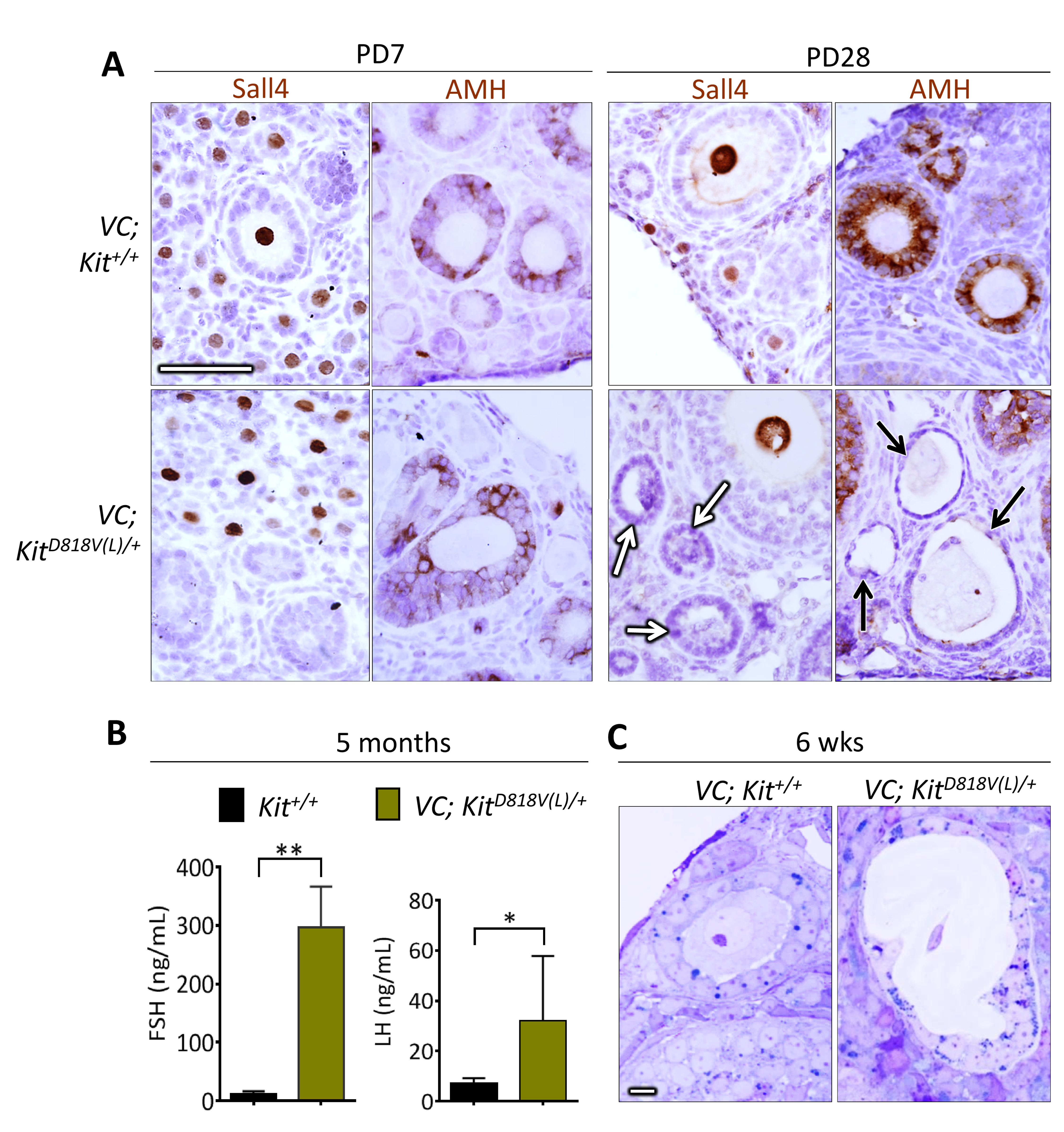

Supplement: S4 Fig — (A) Oocyte reawakening phenotype at PD7 and PD28. Immunohistochemistry for markers as shown; slides counterstained with hematoxylin. White arrows indicate follicles with atretic (Sall4-negative) oocytes; black arrows indicate AMH-negative granulosa cells. Scale bar = 25 μm; all panels at same magnification. (B) Serum FSH and LH levels of VC; KitD818V(L)/+ and control females at five months of age. *p<0.05, **p<0.01; unpaired student t test; n = 3 animals per genotype. (C) Toluidine-blue stained sections of plastic-embedded ovaries from experimental and sibling controls at 6 weeks of age; scale bar = 10 μm, both panels at same magnification. The follicle shown on the right does not contain a viable oocyte. (TIF) [file pgen.1006215.s004.tif]

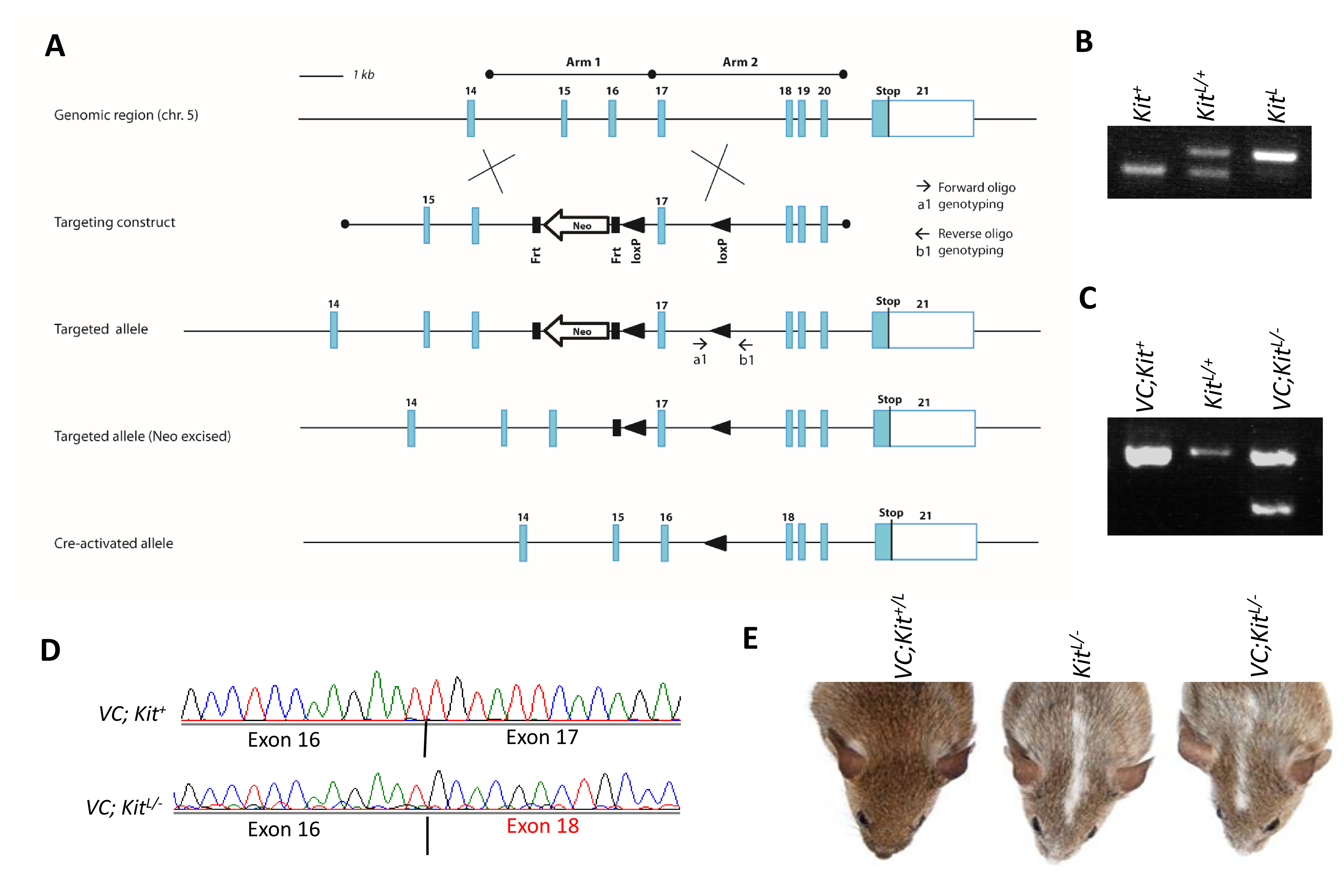

Supplement: S5 Fig — (A) Maps of Kit locus (exons 14–21) and targeting construct. Genotyping primers are indicated by small arrows (a1 and b1). (B) PCR genotyping of wild-type, KitL/+ and KitL/L mouse tails. The 174 bp product corresponds to the KitL allele; the 126 bp product corresponds to the wild-type allele. (C) cDNA expression analysis of mutant allele. cDNA was synthesized from PD3 wild-type and VC; KitL/- ovaries and a region spanning from exon 14 to exon 18 was amplified. The 417 bp (lower) product corresponds to the Cre-mediated exon 17 deletion, while the 545 bp (upper) product corresponds to the wild-type Kit allele. (D) Sanger sequencing of 417 bp cDNA product from VC; KitL/- ovaries confirms precise exon 17 deletion. (E) Mice hemizygous for Kit at 4 weeks of age (but not mice harboring floxed allele, left) show hypopigmentation and midline pigmentation defects. (TIF) [file pgen.1006215.s005.tif]

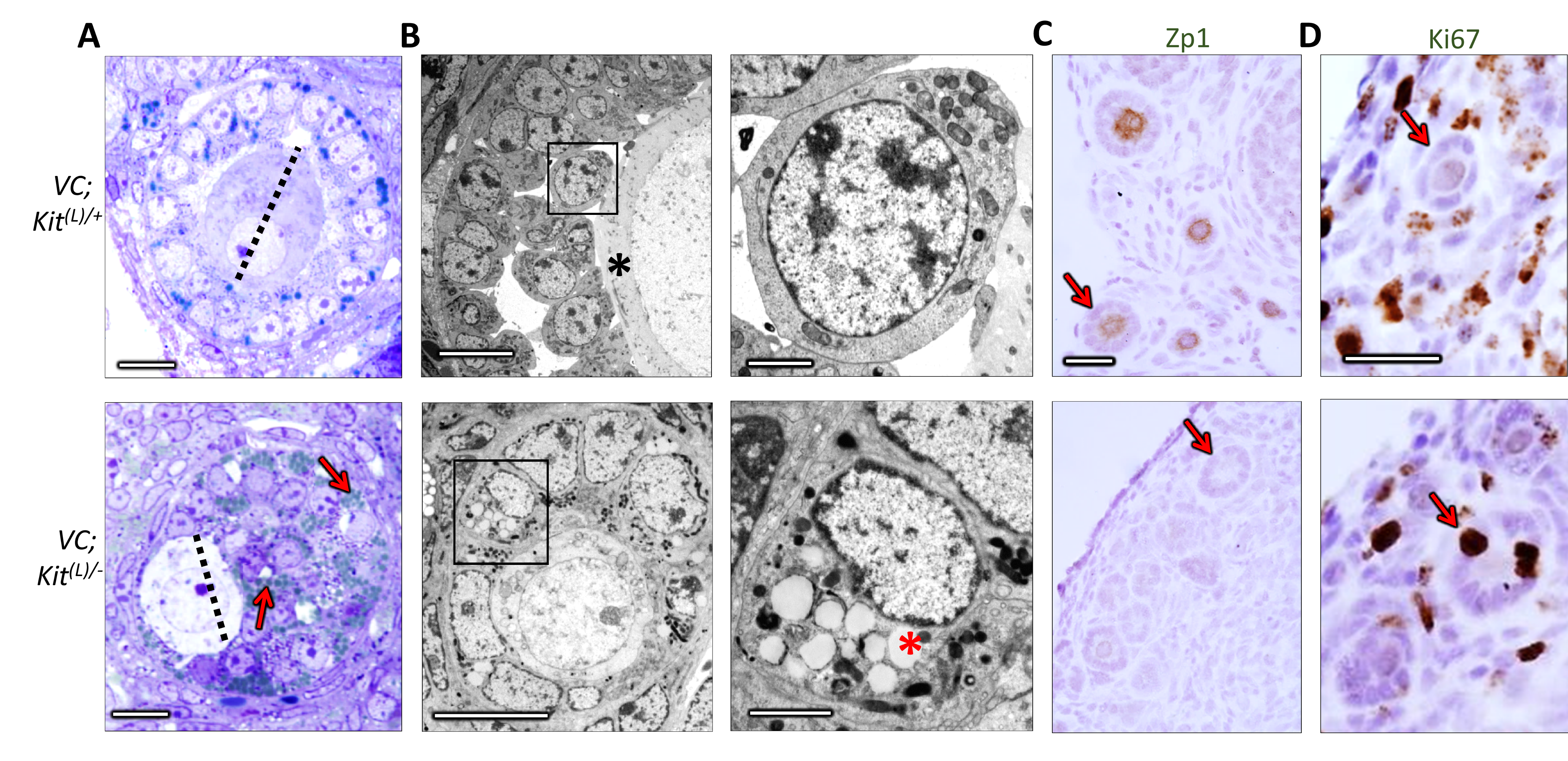

Supplement: S6 Fig — (A) Toluidine-blue stained sections highlight presence of viable but small oocytes that were often eccentrically located within follicles in VC; Kit L/- ovaries. Note lipid droplets in granulosa cells shown by red arrows. Dashed lines demarcate oocyte diameters (30 μm for control, 21 μm for mutant). Scale bars = 10 μm. (B) Transmission electron microscopy of control (top) and VC; Kit L/- ovaries. Note presence of zona pellucida (black asterisk) surrounding a large oocyte in control (left panel). Right panels show high-magnification views of single granulosa cells (indicated by black boxes). In the mutant, note the small, eccentrically-located oocyte and complete absence of a zona pellucida. Higher magnification views of granulosa cells confirm lipid droplets in the mutant (red asterisk). Scale bars = 10 μm for follicle images, 2 μm for granulosa cells. (C) Zp1 immunohistochemistry at 6 weeks of age; slides counterstained with hematoxylin. Red arrows point to small follicles; Zp1 is readily detected in control oocytes. Scale bars = 25 μm, same for both panels. (D) Ki67 immunohistochemistry at 4 weeks of age; slides counterstained with hematoxylin. Red arrows indicate granulosa cells. Scale bars = 25 μm, same for both panels. (TIF) [file pgen.1006215.s006.tif]

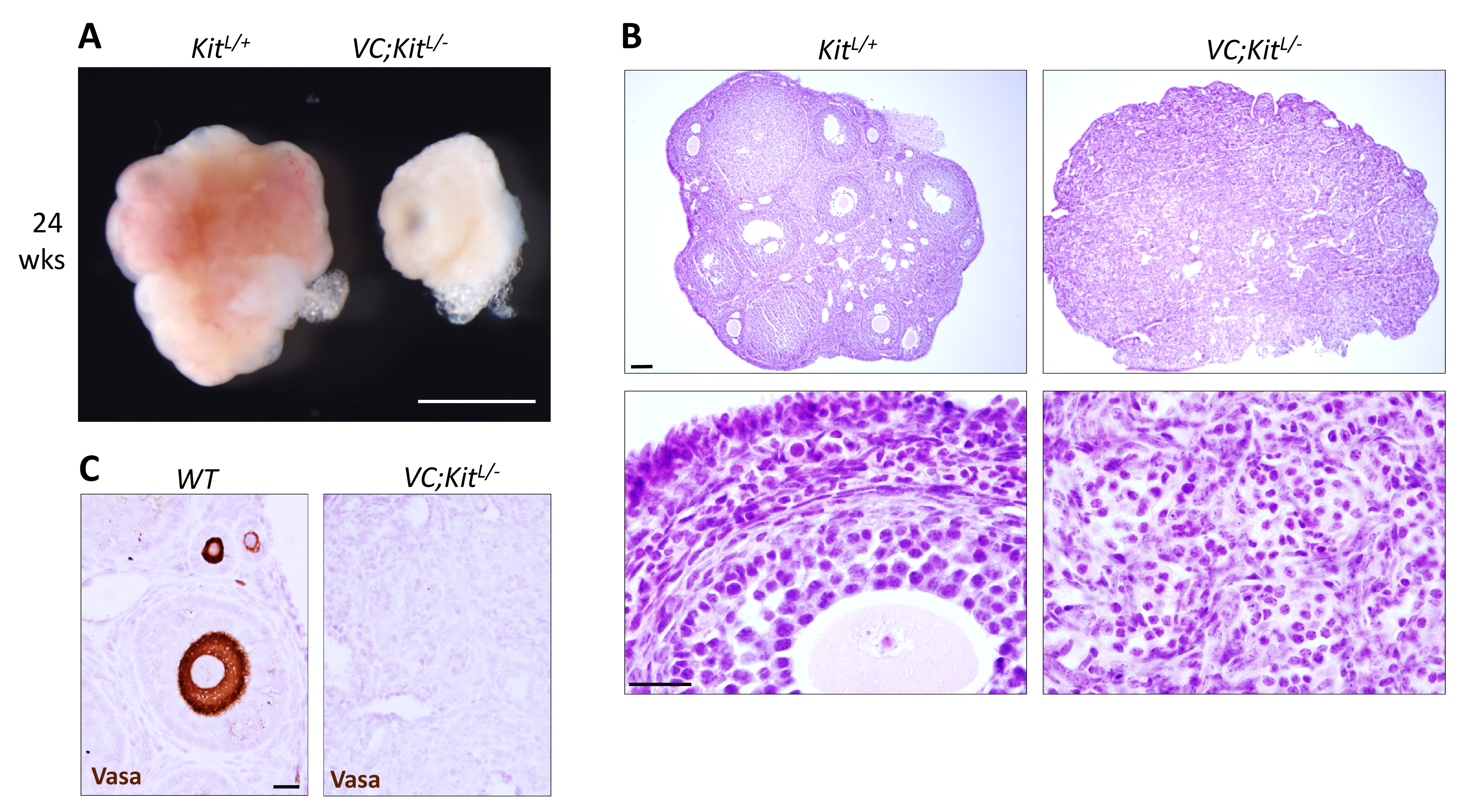

Supplement: S7 Fig — (A) Gross pictures of ovaries from control and VC; KitL/- females. Scale bar = 1 mm. (B) Histological analyses (H&E-stained sections) reveal complete absence of follicles in VC; KitL/- females. Scale bars = 25 μm for all panels. (C) Loss of oocytes by 6 months of age analyzed Vasa immunohistochemistry. Scale bars = 25 μm, same for all panels. (TIF) [file pgen.1006215.s007.tif]
