# Supplementary material for: Control of Oocyte Reawakening by Kit
Source: PLoS Genet. 2016 Aug 8;12(8):e1006215. doi: 10.1371/journal.pgen.1006215 (PMC4976968; doi:10.1371/journal.pgen.1006215)
Supplement: S1 Text — (PDF) [file pgen.1006215.s008.pdf]

Supplementary materials and methods

Generation of *Kit*$^{D818V(L)}$ allele

Arms 1 and 2 (see Figure 1 map) were cloned by PCR from 129S7/SvEv BAC BMQ-452F20 (Source BioScience, Nottingham, UK), which encompasses the *Kit* gene on murine chromosome 5. Arm 2 (4455 bp), which spans exons 17-20, was generated by PCR using primers 5'-GTCTCGAGCACCATAATTTTTATTTTCGGTGTGCTAAA-3' (L) and 5'-GAGGTACCTTACTGAGAGGGATGTCCTAAGTTCAAACA-3' (R). *XhoI* and *KpnI* sites (underlined) plus two random bases to permit efficient digestion were added to the 5' ends of the L and R primers, respectively. Arm 2 PCR was performed with Phusion High Fidelity Taq (NEB, Ipswich, Massachusetts) per the manufacturers' instructions with the following conditions: initial denaturation at 98° x 3 min, followed by 24 cycles of: denaturation at 98° x 1 min and annealing/amplification at 72° x 4 min. Following addition of 3' A overhangs to the gel-purified PCR product, Arm 2 was cloned into pCR2.1-TOPO by TOPO cloning, and one clone was selected for further mutagenesis following sequence confirmation. The pAsp818Val mutation (exon 17) was generated by in vitro mutagenesis (using oligonucleotide 5'-GCGATTTCGGGCTAGCCAGAGTGATCAGGAATGATTCG-3' with the Quickchange II XL Site-Directed Mutagenesis kit (Agilent, Santa Clara, CA) of codon 818 (underlined, with flanking codons shown) from AGA.GAC.ATC.AGG (R-D-I-R) to AGA.GTG.ATC.AGG (R-V-I-R) and also creating a *BclI* (T^GATCA) restriction site polymorphism useful for molecular fingerprinting (e.g. by RT-PCR). Desired site-directed mutagenesis and absence of any undesired mutations was confirmed by Sanger sequencing of the exons in the plasmid. After sequencing, Arm 2 was released from pCR2.1-TOPO by *XhoI* + *KpnI* digestion (NEB). Arm 2 with the engineered mutation was then cloned into the pKOII targeting construct (contains a pGKNeo positive-selection cassette flanked by *frt* sites, a diphtheria toxin negative-selection cassette, and cloning sites for the two homologous recombination arms) (*1*) digested with *XhoI* + *KpnI*.

The allele was designed to provide wild-type *Kit* function prior to Cre-mediated recombination and activation of the allele (*Kit*$^{D818V(L)}$→ *Kit*^{D818V}), a strategy similar to the well-known *Braf*^{V600E} conditional allele (*2*). In the latent *Kit*$^{D818V(L)}$ allele, the C-terminal *Kit* sequences are encoded by an exon 17-21 cDNA/polyadenylation site cassette (shown in green in Figure 1). This 1168 bp cassette was synthesized de novo (DNA 2.0, Menlo Park, CA). In this cassette, the first 151 bp correspond to intron 16-17 sequences, such that the original intronic sequence is reconstituted following Cre-mediated recombination. Also, the majority of wobble positions were modified to limit the likelihood of homologous recombination, which would have resulted in aberrant integration events. This cassette encodes a truncated (139 bp) human 3'UTR to again avoid undesired homologous recombination events (due to the length of the normal 3'UTR, inclusion of the intact 3'UTR sequence was not practicable). *NotI* and *SacII* sites were engineered at the 5' and 3' ends respectively to facilitate cloning in the targeting vector. The precise sequence for this "wobbled" cDNA cassette is available upon request. The cassette was released from the plasmid

vector with *NotI* + *SacII,* and cloned into the *NotI* + *SacII* sites of the pKOII construct harboring Arm 2.

For Arm 1 (2986 bp), which spans exons 15 and 16, the primers for BAC PCR were 5’-GTGCGGCCGCCCCATCATTTTACTGCATTATAGTCACATC-3’ (L) and 5’-GTCGGCCG**A**CAAGTGATATTCCCGTTTTGTGTTTC -3’ (R). *NotI* and *EagI* sites (underlined) plus two random bases to permit efficient digestion were added to the 5’ ends of the L and R primers, respectively. Note: The addition of an extra A residue (bold) before C prevents the *EagI* site from forming an additional *NotI* site following cloning (see below), which would have prevented linearization of the targeting construct. Arm 1 was amplified with the same conditions for Arm 2. Following digestion of the gel-purified PCR product with these enzymes, Arm 1 was cloned into the *NotI* site of pKOII harboring the wobble cassette and Arm 2. Since Arm 1 could be ligated in either orientation, sequencing of several clones was conducted to identify a clone with the Arm 1 insert in the proper orientation. The above strategy results in a targeting construct with a single *NotI* site. The targeting construct was sequenced in its entirety to confirm the desired structure; also, the functionality of the *frt* and *loxP* sites was tested by restriction mapping and Sanger sequencing following transformation into flippase- and cre-expressing *E. coli* (*3*). 300 μg of the targeting construct was linearized with *NotI* and purified. The DNA was extracted with phenol:isoamyl alcohol and ethanol precipitated. The DNA pellet was washed with 70% ethanol and resuspended to a concentration of 1-2 μg/μl.

Electroporation into SM-1 ES cells and subsequent “plus/minus” selection was conducted by standard methods. 576 clones were screened for desired homologous recombination events by PCR screening of Arm 1 and 2. For the screening we used LongAmp Taq PCR kit (NEB). The primers for ES screening were designed to give PCR products only if homologous integration occurred (i.e. no products should arise from wild-type DNA or the unintegrated vector). For Arm 1, the (L) primer is 70 bp outside the 5’ end of the arm, and the (R) primer corresponds to the *Neo* cassette, a sequence absent in mouse gDNA. Similarly, Arm 2 screening primers included (L) corresponding to the *Neo* cassette and an (R) primer 60 bp outside the 3’ end of the arm. The conditions for amplification of Arm 1 (expected product of 4.5 kb) were 94° x 3 min; 94° x 30 sec, 65° x 4 min (34 cycles); 65° x 10 min (primers sequences: 5’ GGCCAAGACCACCCAGCATATGTAGACTAC-3’ (L) and 5’-TCGCCTTCTATCGCCTTCTTGACGAGTTC-3’ (R). The conditions for amplification of Arm 2 (expected product of 4.85 kb) were 94° x 3 min; 94° x 30 sec, 65° x 4 min; 65° x 10 min (primer sequences 5’-CTGACTAGGGGAGGAGTAGAAGGTGGCG-3’ (L) and 5’-TGGTCCCAGTAAACATGGCCAATTCCTCC-3’ (R). Of 14 clones determined to have the desired homologous recombination-mediated targeting event, all were sequenced to confirm the presence of AC to TG mutation in exon 17, and three were selected for blastocyst injection. Chimeric mice were bred with B6(C3)-Tg(Pgk1-FLPo)10Sykr/J transgenic mice (Jackson Laboratory, Bar Harbor, Maine) to excise the *Neo* cassette by *frt*/flippase mediated-recombination and thereby generate the $Kit^{D818V(L)}$ allele.

Generation of *Kit*^{L} allele

The strategy for the generation and linearization of the floxed allele *Kit*^{L} was as above. Arms 1 and 2 were identical, except for the omission of site-directed mutagenesis and inclusion of the wobbled cDNA cassette. pCR2.1-TOPO Arm2 was linearized with *SspI*, which leaves blunt ends. A *loxP* site was integrated into the *SspI* site within the intron following exon 17 by annealing the following oligonucleotides: 5'-ATAACTTCGTATAGCATACATTATACGAAGTTAT-3' (For) and 5'-ATAACTTCGTATAATGTATGCTATACGAAGTTAT-3' (Rev) and ligating the double-stranded *loxP* site into the *SspI* site. After sequencing to confirm integration of the *loxP* site in the desired orientation, Arm 2 was released from pCR2.1-TOPO with *XhoI* + *KpnI* (NEB) and cloned into pKOII digested with the same enzymes. Following digestion with *NotI*, Arm 1 was inserted as previously described. This results (following excision of the *Neo* cassette) in an allele with *loxP* sites flanking exon 17, which encodes the *Kit* tyrosine kinase domain (see Figure 5 schematic). The targeting construct was sequenced in its entirety to confirm the desired structure; also the functionality of the *frt* and *loxP* sites was again tested following transformation into flippase- and cre- expressing *E. coli* (*3*). The targeting construct was linearized with *NotI* and purified as detailed above for the *Kit*$^{D818V(L)}$ allele.

Electroporation into SM-1 ES cells and subsequent "plus/minus" selection was conducted by standard methods. 576 clones (6 x 96 well plates) were screened for desired homologous recombination events by PCR screening of Arm 1 and 2. Screening was performed with the same conditions as for *Kit*$^{D818V(L)}$, with an Arm1 expected product of 3.4kb and an Arm2 expected product of 4.88kb. Of 9 clones determined to have the desired integration, all were sequenced to confirm the presence of *loxP* in the desired location and orientation, and three were selected for blastocyst injection. Chimeric mice were bred with B6(C3)-Tg(Pgk1-FLPo)10Sykr/J transgenic mice (Jackson Laboratory) to eliminate the *Neo* cassette by *frt*/flippase mediated recombination and generate the *Kit*$^{(L)}$ allele.

Supplementary References

1. N. Bardeesy, M. Sinha, A. F. Hezel, S. Signoretti, N. A. Hathaway, N. E. Sharpless, M. Loda, D. R. Carrasco, R. A. DePinho, Loss of the Lkb1 tumour suppressor provokes intestinal polyposis but resistance to transformation. *Nature* **419**, 162-167 (2002).
2. D. Dankort, E. Filenova, M. Collado, M. Serrano, K. Jones, M. McMahon, A new mouse model to explore the initiation, progression, and therapy of BRAFV600E-induced lung tumors. *Genes Dev* **21**, 379-384 (2007).
3. F. Buchholz, P. O. Angrand, A. F. Stewart, A simple assay to determine the functionality of Cre or FLP recombination targets in genomic manipulation constructs. *Nucleic Acids Res* **24**, 3118-3119 (1996).
